# Mesenchymal Stem Cell-Derived Extracellular Vesicles Attenuate Pro-Inflammatory Macrophage Polarization: Comparison of Matrix-Bound and Small Extracellular Vesicles

**DOI:** 10.3390/cells15020093

**Published:** 2026-01-06

**Authors:** Timofey O. Klyucherev, Maria D. Yurkanova, Daria P. Revokatova, Dmitriy A. Chevalier, Vsevolod V. Shishkov, Irina I. Vlasova, Nastasia V. Kosheleva, Peter S. Timashev

**Affiliations:** 1 Institute for Regenerative Medicine, I. M. Sechenov First Moscow State Medical University (Sechenov University), 119991 Moscow, Russia; 2 Laboratory of Clinical Smart Nanotechnologies, Institute for Regenerative Medicine, I. M. Sechenov First Moscow State Medical University (Sechenov University), 119991 Moscow, Russia

**Keywords:** mesenchymal stromal cells, small extracellular vesicles, matrix-bound nanovesicles, monocyte-derived macrophages, phagocytosis

## Abstract

Macrophages play a crucial role in regulating immune responses, inflammation, and tissue repair. Depending on environmental cues, they polarize into pro-inflammatory M1 or anti-inflammatory, pro-regenerative M2 phenotypes. Extracellular vesicles (EVs) derived from mesenchymal stem/stromal cells (MSCs) have emerged as key mediators of intercellular communication and immune modulation. This study investigates the effects of matrix-bound vesicles (MBVs) and small extracellular vesicles (sEVs) derived from human umbilical cord MSCs (UC-MSCs) on human monocyte-derived macrophages (MDMs) in vitro. Both MBVs and sEVs reduced pro-inflammatory activation of M1 macrophages, downregulating the expression of CXCL10 and CD86 while increasing the M2 marker CD206. MBVs exerted a stronger suppressive effect on M1 MDM phenotype markers as well as on STAT1, STAT2, and IRF9 mRNA levels in M1 macrophages, indicating the inhibition of the JAK/STAT1 signaling pathway involved in the pro-inflammatory activation of macrophages. Functionally, both vesicle types enhanced phagocytosis of FITC-labeled *E. coli* by M1 and M0_GM macrophages, promoting a shift toward an M2-like phenotype. Moreover, MBVs and sEVs attenuated reactive oxygen species (ROS) production, with sEVs showing a more pronounced effect both on ROS generation and on the expression of NOX2 complex subunits (p47^phox, p67^phox) in M1 macrophages. These findings demonstrate that MBVs and sEVs from UC-MSCs possess distinct yet complementary immunomodulatory and antioxidant properties on MDMs, suggesting their potential as promising cell-free therapeutic agents for inflammatory and degenerative diseases.

## 1. Introduction

Macrophages are key cells of the innate immune system involved in host defense and the maintenance of tissue homeostasis [1,2]. Depending on endogenous and exogenous stimuli, they can acquire diverse phenotypes. The two most well-characterized states are the pro-inflammatory M1 phenotype and the anti-inflammatory/immunoregulatory M2 phenotype of macrophages [3]. M1 macrophages are involved in inflammation during wound formation and antitumor immunity, and constitute a primary defense against a wide range of pathogens [4]. This phenotype of macrophages is characterized by the increased secretion of pro-inflammatory cytokines, such as IL-1β, IL-6, IL-12, IL-23, and TNF-α, and chemokines CXCL1, CXCL2, CCL3, CCL5, CXCL10, and CXCL11 [5,6]. M1 macrophages also exhibit elevated expression of surface proteins involved in antigen presentation and costimulatory receptors, such as major histocompatibility complex (MHC)-II and the cluster of differentiation (CD)80 and CD86 [4]. Their bactericidal activity relies on the increased production of reactive oxygen species (ROS) via nicotinamide adenine dinucleotide phosphate oxidase (NADPH oxidase, mainly NOX2) and reactive nitrogen species via the enzyme inducible nitric oxide synthase (iNOS) [7,8].

M2 macrophages represent a heterogeneous population including distinct subtypes M2a, M2b, M2c, and M2d, induced in vitro by different cytokines and immunomodulators [9,10,11]. For example, M2a macrophages are induced by IL-4 and IL-13 or in response to fungal and helminth infections [3]. M2 macrophages are characterized by the expression of surface markers such as CD206 (mannose receptor) and CD163 (hemoglobin–haptoglobin receptor), coupled with the secretion of IL-10, transforming growth factor (TGF)-β, and IL-1RA [12,13]. M2 macrophages have a predominantly higher phagocytosing capacity as compared to the M1 phenotype [14]. The excessive accumulation of M1 macrophages is a key driver of chronic inflammation and is implicated in the various diseases such as diabetes, myocardial infarction, lung diseases, and ulcerative colitis [8,15]. Shifting the M1/M2 balance toward alternatively activated M2 macrophages creates an anti-inflammatory microenvironment at the site of injury, promoting tissue repair [16].

Studies of the therapeutic properties of mesenchymal stromal cells (MSCs) in inflammatory and degenerative diseases have shown that MSCs modulate target tissues through anti-inflammatory actions and the stimulation of regeneration, primarily mediated via paracrine signaling and direct cell–cell contacts [17,18,19,20,21,22,23]. MSCs promote the phenotypic switch of M1 macrophages toward M2 macrophages, thereby facilitating the resolution of inflammation and tissue regeneration [22,23,24,25]. The immunomodulatory properties of MSCs, including the enhancement of macrophage polarization into the M2 phenotype and increasing the proportion of T-regulatory cells, have a positive effect on reducing degenerative processes for immune-mediated and inflammatory diseases, including osteoarthritis [22,23]. MSC-based therapy is considered relatively safe, but concerns remain regarding potential immunogenicity, as well as the risks of thrombosis and tumorigenic transformation following in vivo administration of MSC [26,27].

As an alternative to MSC-based cell therapy, components of their secretome, such as secreted cytokines, growth factors, and extracellular vesicles (EVs), are actively studied [27,28,29,30]. MSC-derived EVs exert predominantly immunosuppressive effects on macrophages and other immune cells [30]. Several classes of EVs can be distinguished, including exosomes, microvesicles, apoptotic bodies, and the recently described matrix-bound nanovesicles (MBVs) [31,32,33,34]. MSC-derived EVs demonstrate high therapeutic potential in vitro and in vivo, with one of their anti-inflammatory effects attributed to their ability to promote macrophage polarization toward the M2 phenotype [35]. For example, EVs derived from MSCs obtained from infrapatellar fat pad tissue, genetically modified to express an antagonist of calcitonin gene-related peptide, were shown to modulate pain and inflammation by promoting the transition of M1 macrophages toward the M2 phenotype and attenuating the neuroinflammatory profile of cortical neurons, which has therapeutic potential for reducing inflammation and pain, including in osteoarthritis [36]. In another study, MSCs generated from induced peripheral blood fibroblasts produced EVs enriched in four microRNAs (hsa-miR-142-3p, hsa-miR-146a, hsa-miR-107, hsa-miR-25-3p), which regulate the immune system and inflammation by modulating excessive pro-inflammatory macrophage responses and cytokine secretion [29]. The effects of these EVs were demonstrated in vitro in THP-1 macrophages, where EVs promoted a phenotypic shift from the M1 to the M2 state, which was also confirmed in vivo in a model of acute synovial inflammation induced by monoiodoacetate [29]. EVs derived from human umbilical cord-derived MSCs (UC-MSCs) enhanced motor function recovery in vivo by increasing the local abundance of M2 macrophages, accompanied by a reduction in inflammatory cytokines tumor necrosis factor-α TNF-α, IL-6, and interferon (IFN)-γ [37]. Researchers commonly use the term “small EV” for vesicles smaller than 100 or 200 nm and “medium/large EV” for vesicles larger than 200 nm [38]. The size ranges of different EV types are 50–1000 nm for microvesicles, 30–150 nm for exosomes, 20 to 400 nm for MBVs, and 50–5000 nm for apoptotic bodies [39]. Due to size overlap, populations of EVs isolated by size are often heterogeneous, requiring careful use of terminology. The International Society for Extracellular Vesicles (ISEV) 2023 guidelines recommends using the general term “extracellular vesicles” instead of “exosomes” to designate small EVs (sEVs) if selective methods were applied that cannot isolate only exosomes from fluid samples [38]. Based on this, the present study uses the term sEVs for EVs derived from UC-MSC conditioned medium, as differential ultracentrifugation may co-isolate microvesicles around 200 nm that overlap with exosomes and other sEVs.

MBVs represent a recently identified subpopulation of EVs, and several studies have demonstrated their regenerative potential, which is primarily based on their immunomodulatory effects and ability to modulate the M1/M2 macrophage balance [40]. MBVs are components of the extracellular matrix (ECM) of soft tissues, monolayer cell cultures, and spheroids [34,41]. In contrast to fluid-phase EVs such as exosomes, MBVs exhibit lower expression on canonical tetrapanins CD63, CD81, and CD9, and their protein composition differs from the other EVs. This suggests that MBVs are a distinct subpopulation of nanovesicles, whose biological activity is manifested during ECM degradation following tissue injury [42]. MBVs derived from small intestine mucosa and urinary bladder matrix exerted immunomodulatory effects on murine bone marrow-derived M1 macrophages by reducing iNOS and TNF-α expression, increasing arginase-1 expression, and enhancing the phagocytic capacity of M1 macrophages after MBV exposure, indicating a shift in M1 macrophages toward an M2-like phenotype [43]. In addition to MBVs from soft tissue ECM, MBVs obtained from UC-MSC monolayer cultures reduced TNF-α and IL-6 expression in human M1 macrophages [34]. Despite these promising findings, a comprehensive comparison of the effects of MBVs and small EVs (exosomes) derived from MSCs on various aspects of functional activity of human peripheral blood monocyte-derived macrophages is lacking. In this study, we aimed to compare the effects of MBVs and sEVs derived from human UC-MSCs on M1 polarization markers, surface receptors, ROS production, phagocytic activity, and mRNA expression of NOX2 subunits, intracellular signaling molecules for four MDM phenotypes: M0_GM, M1, M0_M, and M2.

## 2. Materials and Methods

### 2.1. Source of UC-MSCs and Cultivation of Human UC-MSC Monolayer Cultures for EV Isolation

Human MSCs were obtained from the biobank of the Institute for Regenerative Medicine, Biomedical Science and Technology Park, Sechenov University (I.M. Sechenov First Moscow State Medical University, Ministry of Health of Russia). Cells were extracted from the Wharton’s jelly in the umbilical cord. To isolate MSCs, umbilical cord samples were obtained from *n* = 6 donors. In accordance with the Declaration of Helsinki, tissue collection was performed after obtaining informed donor consent approved by the local ethics committee (No. 16–21, 16 September 2021, Moscow, Russia). Biopsies were processed under sterile conditions in a class II biological safety cabinet within 2 h of collection. MSC isolation and culture were performed following the protocol described by Peshkova et al., 2023 [44].

### 2.2. Analysis of Specific Multipotent MSC Markers by Flow Cytometry

UC-MSC immunophenotyping was performed according to the protocol described in Klyucherev et al. [34]. Cells were assessed for surface positivity of CD90, CD73, CD105, and CD44, along with the absence of CD34, CD11b, CD19, and CD45 expression (hMSC Analysis Kit, BD Stemflow™, San Diego, CA, USA). Data acquisition was performed using a Sony SH800 microfluidic cell sorter (Sony Biotechnology, San Jose, CA, USA). Unstained and isotype controls were included to ensure antibody specificity.

### 2.3. Isolation of Matrix-Bound Vesicles and Extracellular Vesicles from Conditioned Medium of Multipotent Mesenchymal Stromal Cells

Forty-eight hours before EV isolation, Petri dishes with monolayer MSC cultures were washed three times with Hank’s balanced salt solution to remove residual EVs and proteins contained in FBS, and then cultured in serum-free DMEM/F12 medium supplemented with 2 mM L-glutamine (Biolot, Novosibirsk, Russia). For MBV isolation from monolayer cell cultures, conditioned medium from Petri dishes was collected for subsequent sEV isolation, and the surface of Petri dishes with monolayer MSCs was washed three times with sterile PBS to remove residual conditioned medium. For MBV isolation, extracellular matrix digestion was carried out by adding 1 mL of an enzymatic mixture to each Petri dish, consisting of collagenase I (2 mg/mL, Gibco™, 17100017, Grand Island, NY, USA), dispase (1.5 U/mL, Gibco™, 17105041, Grand Island, NY, USA), and collagenase II (1 mg/mL, Gibco™, 17101015, Grand Island, NY, USA). The dishes were then incubated with the proteases for 60 min at 37 °C. Following enzymatic digestion, the ECM fraction intended for MBV isolation and the MSC-conditioned medium containing sEVs were placed into separate centrifuge tubes. Samples were first spun at 400× *g* for 10 min, after which the resulting supernatants were further centrifuged at 2500× *g* for 20 min. Subsequently, the clarified supernatants containing MBVs and sEVs were transferred to ultracentrifuge tubes and subjected to centrifugation at 10,000× *g* for 30 min at 4 °C. The final supernatants were then filtered through 0.22 µm PES syringe filters (ALWSCI Technologies, Shaoxing, China). Supernatants of sEVs and MBVs collected from centrifuge tubes were passed through 0.22 µm PES syringe filters (ALWSCI Technologies, Shaoxing, China). EVs were subjected to two rounds of ultracentrifugation at 120,000× *g* for 90 min at 4 °C using an Optima XPN-100 ultracentrifuge (Beckman Coulter, Indianapolis, IN, USA). Following centrifugation, the supernatant was removed, and the resulting pellet was resuspended in sterile PBS, passed through a 0.22 µm PES syringe filter, and stored at −80 °C.

### 2.4. Morphology and Size Assessment of Matrix-Bound Vesicles and Extracellular Vesicles Derived from Conditioned Medium of Human Umbilical Cord Mesenchymal Stromal Cells

Total vesicle protein content was quantified using a QuantiPro™ BCA assay kits (Sigma-Aldrich Co. LLC, Burlington, MA, USA). Vesicle morphology was examined by transmission electron microscopy (TEM) following negative staining with uranyl acetate. Morphology was analyzed using a JEM-1011 TEM (Jeol, Tokyo, Japan). Average sizes of MBVs and sEVs were determined with a Zetasizer Nano ZS (Malvern Panalytical, Malvern, UK) following the method in [41].

### 2.5. Western Blot Analysis of Vesicle Markers

The expression of exosomal markers in MBV and sEV samples was analyzed by SDS-PAGE followed by Western blotting. To confirm the absence of intracellular contaminants, UC-MSCs (passage 4) were lysed in RIPA buffer with protease–phosphatase inhibitors on ice for 10 min, debris removed by centrifugation (10,000× *g*, 10 min), and MSC lysates stored at −80 °C. Equal amounts of protein (15 µg per lane) were mixed with Laemmli sample buffer, heat-denatured, and separated on 12% SDS–polyacrylamide gels. Proteins were transferred onto 0.2 µm nitrocellulose membranes using a semi-dry blotting system. Membranes were blocked with 5% BSA in TBST for 1 h, incubated overnight with primary antibodies: CD9 (1:1000, Abcam ab223052, Waltham, MA, USA), CD63 (1:1000, Abcam ab68418, Waltham, MA, USA), TSG101 dilution 1:5000 (Abcam, ab125011, Waltham, MA, USA), Calnexin (1:1000, Abcam ab10286, Waltham, MA, USA), and Glyceraldehyde-3-phosphate dehydrogenase (GAPDH) (1:2000, Cloud-Clone CAB932Hu22, Wuhan, China). After TBST washes, membranes were incubated with HRP-conjugated anti-rabbit IgG secondary antibodies (1:10,000, Arigobio, Hangzhou, China). Protein signals were visualized using ECL detection reagents (Thermo Fisher, Waltham, MA, USA) and imaged with the iBright CL1500 system (Invitrogen, Carlsbad, CA, USA).

### 2.6. PKH26 Labeling of sEVs and MBVs

Freshly isolated MBVs and sEVs were labeled with the PKH26 membrane dye (Lumiprobe, Moscow, Russia) according to manufacturer’s protocol. MBVs and sEVs were diluted to 1 mL with Diluent C, mixed with 6 µL PKH26, resuspended for 30 s, and incubated 5 min. A dye-only control was prepared identically without EVs. Excess dye was quenched with 2 mL 10% BSA, diluted to 10 mL PBS, and removed by ultracentrifugation (120,000× *g*, 70 min, 2–8 °C). EV pellets were resuspended in PBS and further purified using Amicon 50 kDa filters (Millipore, Sigma-Aldrich Co. LLC, Burlington, MA, USA) with three PBS wash–concentration cycles. For confocal imaging, 10 µL of fluorescently labeled MBVs and sEVs were placed onto poly-L-lysine–coated slides, allowed to air-dry for 30 min in the absence of light, and mounted with ImmunoHistoMount (Abcam, Waltham, MA, USA). The samples were then examined using an Olympus FV3000 confocal microscope (Tokyo, Japan) equipped with a 100×/1.40 oil immersion objective and 568 nm excitation.

### 2.7. Differentiation and Polarization of Monocyte-Derived Macrophages from Human Monocytes

**Figure 1:**
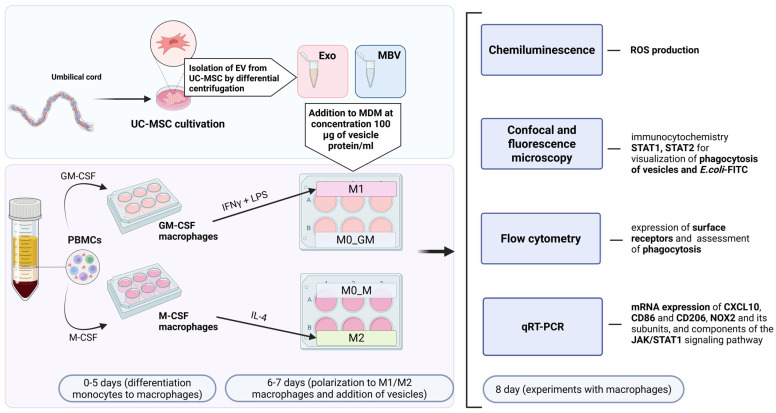
Scheme of the experiments with MDMs supplemented with MBVs and sEVs obtained from UC-MSCs.

Macrophages were cultured followed Klyucherev et al. [34]. Peripheral blood mononuclear cells were isolated from blood of healthy donors (18–60 years, BMI 18.5–24.9, number of donors *n* = 18) and plated at a density of 7.5–8.5 × 10^5^ PBMCs/cm^2^. Differentiation of monocytes into macrophages was induced with granulocyte-macrophage colony-stimulating factor (GM-CSF) or macrophage colony-stimulating factor (M-CSF) (50 ng/mL, SCI-store, Moscow, Russia), with media changes on days 3 and 6. Unpolarized macrophages (M0_GM, M0_M) were retained. M1 polarization was induced with LPS (10 ng/mL) + IFN-γ (50 ng/mL), M2 with IL-4 (25 ng/mL) added on day 6 of incubation. MBVs or sEVs were added at a concentration of 100 µg/mL protein following polarization. Cells and supernatants were collected after 48 h for downstream analyses. As part of each test to analyze the effect of EV UC-MSCs on the polarization and functional activity of MDMs, macrophages were used, where *n* is a separate PBMC donor. The general design scheme of the experiment is demonstrated in (Figure 1).

### 2.8. Phagocytosis of PKH26-Labeled MBV and sEV UC-MSCs by Macrophages

PKH26-labeled MBVs and sEVs were added to M1 macrophages (LPS + IFN-γ) at 100 µg/mL protein. Controls: (i) free PKH26 dye, (ii) unlabeled sEVs, (iii) cells were incubated 12 h at 37 °C. Nuclei were stained with Hoechst 33,258 (0.004 mg/mL) 30 min before fixation. Cells were washed with PBS and fixed with 4% paraformaldehyde, washed with PBS, and analyzed on Olympus FV3000 confocal microscope (100×/1.40 oil). Excitation: Hoechst 405 nm, PKH26 568 nm; detection: Hoechst 430–480 nm, PKH26 580–620 nm.

### 2.9. Quantitative Polymerase Chain Reaction with Reverse Transcription

Total RNA was extracted from macrophages (~2.8–3.2 × 10^5^ cells/sample) using RUplus-250 kit (Biolabmix, Novosibirsk, Russia). Purity/concentration were measured on Nanodrop 8000. Genomic DNA was removed with DNase E, samples purified with CleanRNA Standard kit (Evrogen, Moscow, Russia). Reverse transcription: 1 µg RNA with MMLV RT (Evrogen, Moscow, Russia). qPCR was run with 50 ng cDNA and qPCRmix-HS SYBR (Evrogen, Moscow, Russia). Primers were designed using Primer-BLAST (https://www.ncbi.nlm.nih.gov/tools/primer-blast/, accessed on 21 December 2025) GAPDH: fwd GCACCGTCAAGGCTGAGAAC, rev CCACTTGATTTTGGAGGGATCT; CD206: fwd CCAGACACGATCCGACCCTT, rev AGCTTGCAGTATGTCTCCGCT; CD86: fwd GAGTGAACAGACCAAGAAAAGAGAA, rev AAAAACACGCTGGGCTTCATC; CXCL10: fwd GCAGTTAGCAAGGAAAGGTCT, rev CCTCTGTGTGGTCCATCCTT; p47phox: fwd GTACCGCGACAGACATCACC, rev ACCGCTCTCGCTCTTCTCTA; p67phox: fwd AGCGGACAAGAAGGACTGGA, rev TCCCTCGTTGGAAGTAAGCC; NOX2 (CYBB): fwd AACTGGGCTGTGAATGAGGG, rev GCCAGTGCTGACCCAAGAA; IRF9: fwd TTGCCCGATACTTGCTGGAG, rev AGGTGAGGTGGAAGATGGGT; STAT2: fwd AAACTCCCCATCGACCCCTC, rev GTCTCACCAGCAGCCTTGTTC; STAT1: fwd GGCCAAAGGAAGCACCAGAGC, rev TACAGAGCCCACTATCCGAGACACC. Quantitative reverse transcription polymerase chain reaction (qRT-PCR) conditions were as follows: initial denaturation at 95 °C for 3 min, followed by 40 amplification cycles consisting of denaturation at 95 °C for 15 s, annealing at 60 °C for 15 s, and extension at 70 °C for 20 s. Relative transcript levels were calculated using the ΔΔCt approach and normalized to GAPDH expression (*n* = 2^−ΔC×1000^, where ΔC = C1 − Chk, with C1 representing the target gene and Chk the GAPDH reference gene).

### 2.10. Measurement of Macrophage Polarization Surface Markers by Flow Cytometry

On the 6th day of cultivation, macrophages were exposed to extracellular vesicles at a final concentration of 100 µg protein/mL. After 48 h of incubation at 37 °C, the cells were detached using Accutase solution (Sigma-Aldrich, Burlington, MA, USA) for 5–7 min at 37 °C under 5% CO_2_ to generate a single-cell suspension. The suspension was diluted with complete RPMI medium (volume ratio 1:8) and centrifuged at 300× *g* for 7 min. The cell pellets were rinsed twice with PBS to eliminate remaining Accutase and subsequently resuspended in PBS supplemented with 1% FBS. The cells were divided into aliquots and incubated with antibodies against CD206-PE-Cy7 or CD86-FITC (Invitrogen, Carlsbad, CA, USA) according to the manufacturer’s protocol. After washing, samples were analyzed using a Sony SH800 microfluidic cell sorter (Sony Biotechnology, USA), with a minimum of 10,000 events recorded for each sample. Data processing and analysis were performed using FlowJo software (v10), with appropriate gating strategies applied to exclude debris and doublets.

### 2.11. Immunohistochemical Staining

For immunofluorescence analysis, M1 macrophages were incubated with UC-MSC–derived vesicles for 48 h. Cells were washed with PBS and incubated with Hoechst nuclear stain prior to fixation. Fixation was performed using 4% paraformaldehyde at 4 °C, followed by permeabilization with 0.1% Triton X-100 at RT. After that, the samples were washed 3 times in PBS-Tween 0.05% solution. The samples were blocked in 3% BSA for 1 h at room temperature. After that, primary antibody STAT1 at a dilution of 1:200 (PAB740Hu01, Cloud-Clone, Wuhan, China) and STAT2 at a dilution of 1:200 (PAB796Hu01, Cloud-Clone, Wuhan, China) were added, and the samples were incubated overnight at +4 °C. After that, the samples were washed 3 times in PBS-Tween 0.05% solution. Anti-Rabbit IgG superclonal recombinant secondary antibody, Alexa Fluor 488 (Invitrogen, Carlsbad, CA, USA) at a dilution of 1:1000 was added to the samples and the samples were incubated at room temperature for 60 min in the dark. In addition to the experimental groups, the following controls were performed: secondary antibody Alexa Fluor 488 without primary antibody, negative control without antibodies. After that, the samples were washed 3 times in PBS-Tween 0.05% solution. The cells were analyzed using an EVOS M5000 fluorescence microscope (Thermo Fisher Scientific, Waltham, MA, USA). Hoechst-stained nuclei were visualized using excitation at 350–360 nm and emission at 440–480 nm, while Alexa Fluor 488 fluorescence was detected using excitation at 470–495 nm and emission at 515–525 nm.

### 2.12. Analysis of the Effect of EV UC-MSCs on Macrophage Phagocytic Activity by Flow Cytometry and Fluorescence Microscopy

Macrophages were maintained in 48-well plates for fluorescence imaging and in 12-well plates for flow cytometry experiments. Phagocytosis was evaluated on the eighth day of differentiation using FITC-conjugated *Escherichia coli* (*E. coli*) bioparticles (Molecular Probes, Eugene, OR, USA). Prior to use, the bioparticles were resuspended in complete RPMI medium and added to cells at a defined bacteria-to-macrophage ratio. The cultures were incubated for 1–2 h at 37 °C under 5% CO_2_, after which non-internalized particles were removed by washing the cells three times with PBS. The cells were then incubated with Accutase for 5 min and detached using cell scrapers. The obtained cell suspension was centrifuged at 300× *g* for 10 min. The cell pellets were resuspended in ice-cold solution to stop the reaction and incubated for 5 min. Following centrifugation, the cells were suspended in a fixation solution consisting of PBS supplemented with 0.5% FBS and 1% paraformaldehyde. After a 20 min fixation period, the cells were pelleted again and reconstituted in flow cytometry buffer (PBS containing 1% FBS). Fluorescence signals were acquired on a Sony SH800 cell sorter (Sony Biotechnology, USA) and subsequently processed using FlowJo software (v.10).

To assess phagocytosis by fluorescence microscopy, macrophages were first incubated with *E. coli* bioparticles and then exposed to an ice-cold quenching solution to suppress fluorescence from non-internalized particles. Cells were fixed in 4% paraformaldehyde at 4 °C for 20 min and subsequently permeabilized with 0.1% Triton X-100 for 5 min at room temperature. The actin cytoskeleton was labeled using rhodamine–phalloidin (Thermo Fisher Scientific, Waltham, MA, USA), with samples incubated for 2 h in the dark, followed by nuclear staining with DAPI (Thermo Fisher Scientific) for 20 min between each step, and cells were rinsed three times with PBS. Imaging was performed using an EVOS M5000 fluorescence microscope (Thermo Fisher Scientific, Waltham, MA, USA). For visualization of nuclei stained with DAPI, an LED excitation source with a wavelength of ~360 nm was used, and the signal was detected in the range of 440–480 nm. For visualization of actin filaments stained with rhodamine–phalloidin, an excitation range of 530–550 nm and emission range of 570–620 nm were used. Internalized *E. coli*-FITC particles were detected at excitation 470–495 nm and emission 510–540 nm.

### 2.13. Luminol-Dependent Chemiluminescence

Reactive oxygen species generation by MDMs was determined using the luminol-dependent chemiluminescence (CL) method. Macrophages were cultured in 12-well plates, washed twice with Versene solution to remove the medium and calcium ions, then incubated for 7 min with Accutase solution (Sigma-Aldrich, Burlington, MA, USA USA) and collected using a cell scraper. After two centrifugations in complete medium (to remove Accutase) and PBS (to remove medium residues), the cells were counted and diluted in Krebs–Ringer solution with NaHCO_3_ and CaCl_2_ (pH 7.4) to a concentration of 1 × 10^6^ cells/mL. The reaction mixture contained 30 µg/mL horseradish peroxidase and 200 µM luminol. To activate reactive oxygen species production, 100 ng/mL phorbol 12-myristate 13-acetate (PMA, Sigma-Aldrich, Co. LLC, Burlington, MA, USA) was added. Luminescence intensity was recorded immediately after PMA addition using a Lum-1200 chemiluminometer (DISoft, Moscow, Russia). Gentle shaking was performed between measurement cycles. Areas under chemiluminescence curves (AUCs) were compared for analysis.

### 2.14. Statistical Analysis

Statistical analysis was conducted using GraphPad Prism version 10.3 for Windows (GraphPad Software (v10.3), San Diego, CA, USA). The Shapiro–Wilk test was employed to assess normality of data distribution. Based on these results, either the Kruskal–Wallis test or one-way ANOVA with suitable post hoc comparisons was applied. Differences were considered statistically significant at *p* ≤ 0.05. Data are presented as box-and-whisker plots showing median values.

## 3. Results

### 3.1. Immunophenotyping of UC-MSCs; Characterization of Size, Morphology, and Expression of Exosomal Markers of sEV and MBV UC-MSCs

**Figure 2:**
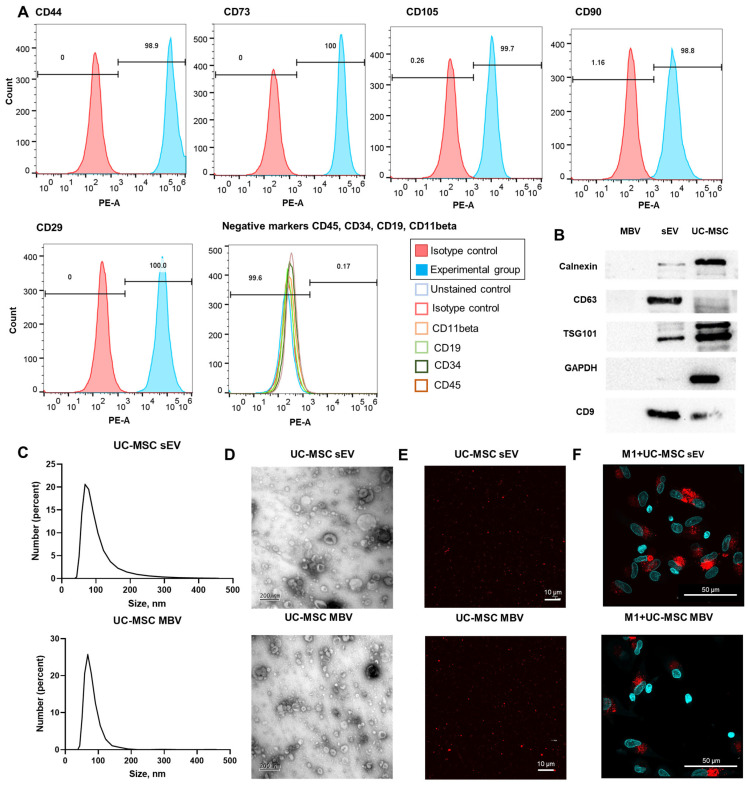
Characterization of UC-MSC and UC-MSC-derived vesicles (sEVs, MBVs). (**A**) Flow cytometry analysis of UC-MSCs of passage 4 demonstrated the expression of phenotypic markers of MSCs (CD44, CD73, CD105, CD90, CD29) and absence of hematopoietic markers on cell surface (CD19, CD11β, CD45, CD34). (**B**) Western blotting of the proteins of UC-MSC vesicles (MBVs and sEVs) and UC-MSC lysate for the presence of positive exosomal markers (CD9+, CD63+, TSG101+) and negative ones (GAPDH-, Calnexin-). (**C**) Particle size distribution obtained by dynamic light scattering analysis of nanoparticles. (**D**) Visualization of sEVs and MBVs obtained from UC-MSCs using TEM. Scale bar 200 nm. Magnification ×60,000. (**E**) Confocal microscopy images of vesicles obtained from UC-MSCs (sEVs and MBVs) labeled with the fluorophore dye PKH26. Scale bar 10 µm. Magnification ×100. (**F**) Microphotographs of M1 macrophages that phagocytosed EVs labeled with the membrane dye PKH26. Red fluorescence indicates membrane dye PKH26, and blue fluorescence indicates nuclear dye Hoechst 33258. Magnification ×100. Scale bar 50 µm.

Flow cytometry demonstrated that UC-MSCs express characteristic mesenchymal markers (CD44, CD73, CD105, CD90, CD29) with negligible expression of hematopoietic markers (CD19, CD11beta, CD45, CD34) (Figure 2A). Positive mesenchymal markers included CD44 (98.9%), CD73 (100%), CD105 (99.7%), CD90 (98.8%), and CD29 (100%) for UC-MSCs, while the proportion of negative markers did not exceed 0.17%. The isolated sEVs expressed characteristic exosomal markers CD9+, CD63+, TSG101+ (Figure 2B). In contrast, MBVs did not express detectable levels of exosomal markers (Figure 2B). An analysis of positive and negative vesicle markers in UC-MSC lysates was also performed (Figure 2B). The results showed that UC-MSCs had less pronounced expression of positive vesicle markers such as CD9+, CD63+, while these cells exhibited a higher expression of GAPDH and Calnexin compared to vesicles of both types. This suggests that vesicle samples were not significantly contaminated with intracellular proteins such as the cytosolic protein GAPDH and Calnexin found in the endoplasmic reticulum of cells (Figure 2B). Dynamic light scattering analysis of nanoparticles showed that the obtained sEV and MBV UC-MSCs had a size range of 40–150 nm, with the size of MBV UC-MSCs being 74.3 ± 19.4 nm (mean ± standard deviation), and sEV UC-MSCs being slightly larger—89.6 ± 40.6 nm (Figure 2C). TEM images showed that MBVs and sEVs have a characteristic cup-shaped rounded morphology and a particle size range of 50–200 nm (Figure 2D). Confocal microscopy demonstrated that both types of vesicles obtained from UC-MSCs contain lipid bilayers, as they are capable of adsorbing the cyanine dye PKH26, which non-covalently integrates into lipid membranes. It is known that lipid membranes are the structural basis of membrane nanoparticles such as MSC-derived vesicles (Figure 2E). More detailed information is provided in Supplementary Materials (Figures S1–S3).

### 3.2. Phagocytosis of EV UC-MSCs by M1 MDMs and Modulation of Macrophage Polarization Markers After Treatment with EV UC-MSCs

Macrophages derived from peripheral blood mononuclear cells were chosen as an in vitro model to evaluate the immunomodulatory properties of sEV and MBV UC-MSCs. The ability of these extracellular vesicles to modulate the pro-inflammatory activity of MDMs was assessed for four phenotypes: M0 cultured with GM-CSF (M0_GM), M0 cultured with M-CSF (M0_M), pro-inflammatory M1 macrophages (M0_GM + (IFNγ + LPS)), and reparative M2 macrophages (M0_M + IL-4). The ability of macrophages to phagocyte vesicles was demonstrated for sEVs and MBVs labeled with the membrane dye PKH26 using M1 macrophages as an example (Figure 2F). For both types of PKH26-labeled vesicles, intense fluorescence corresponding to phagocytosed dye-labeled vesicles was observed (Figure 2F). To assess whether residual unbound PKH26 dye present in vesicle preparations could be phagocytosed, M1 macrophages were cultured with this dye precipitated without vesicles. The results showed weak phagocytosis of the dye (Figure S4). The fluorescence intensity of the cells was significantly lower than that in M1 samples incubated with PKH26-labeled sEVs and MBVs (Figure S4). These results suggest that PKH26-labeled vesicles were predominantly purified from the dye, and the intracellular phagosomes showing red fluorescence were due to phagocytosis of PKH26-labeled vesicles rather than free PKH26 dye or its micelles. To control the effect of unlabeled vesicles, sEVs without PKH26 labeling were added to M1 macrophages. No PKH26 signal was observed in microphotographs (Figure S4).

**Figure 3:**
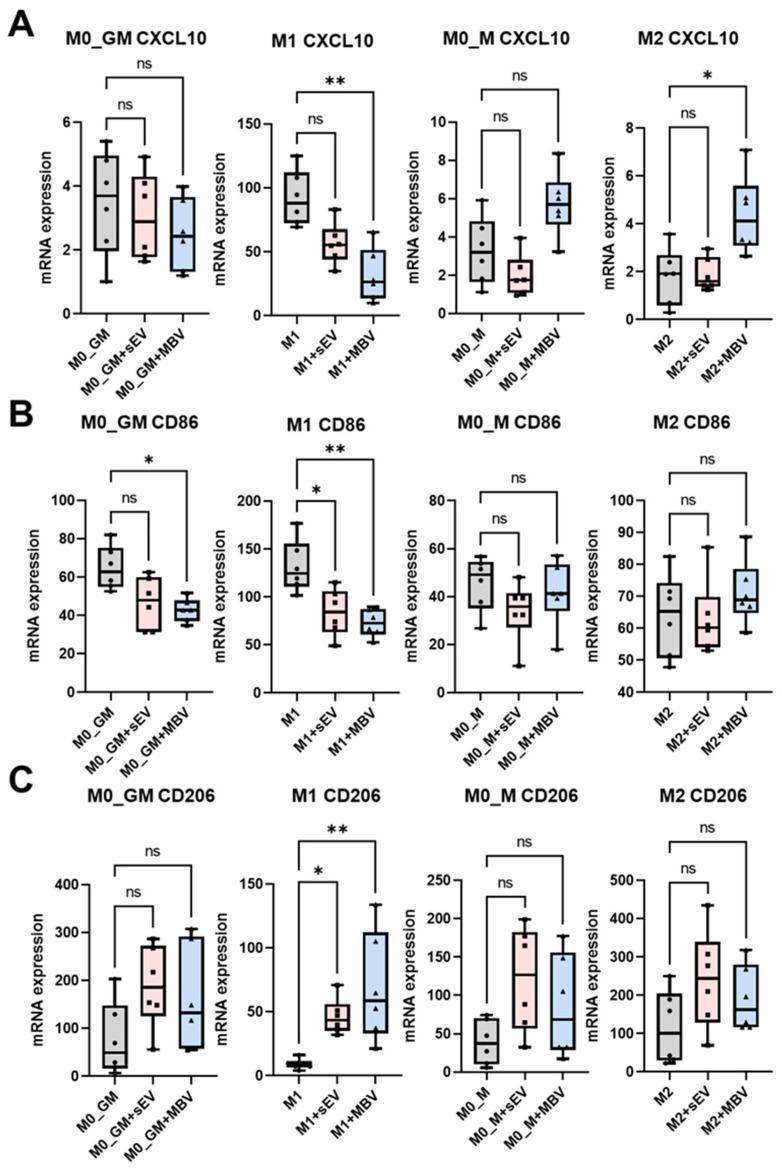
Modulation of mRNA expression of CXCL10 chemokine and surface macrophage polarization markers CD86, CD206 in four MDM phenotypes after incubation with sEVs or MBVs obtained from UC-MSCs. (**A**) qRT-PCR for CXCL10, (**B**) qRT-PCR for CD86, (**C**) qRT-PCR for CD206. The number of MDM donors is *n* = 6. The Kruskal–Wallis nonparametric test followed by Dunn’s multiple comparison post-test was used for statistical analysis. This test was used for each experimental group. * *p* < 0.05; ** *p* < 0.01; ns (*p* > 0.05).

In this study, the effect of EV UC-MSCs on the expression of MDM surface receptors (CD86, CD206), the chemokine motif ligand (CXCL)10, and components of the JAK/STAT1 signaling pathway were analyzed (Figure 3). In our previous study, EV UC-MSCs were added to MDMs, and an immunosuppressive effect of sEVs and MBVs on M1 macrophages was demonstrated, where both types of vesicles reduced the expression and secretion levels of TNF-α and IL-6 mRNA and proteins [34]. For EV dose normalization, protein-based quantification was used for both sEV and MBV UC-MSC preparations. Both EV types at a concentration of protein 100 μg/mL and 300 μg/mL significantly reduced TNF-α secretion in M1 macrophages (Supplementary Materials, Figure S5). Since no substantial difference was observed between these concentrations, subsequent experiments were performed using 100 μg/mL. This concentration is widely used in MSC-EV macrophage studies in vitro [45,46,47]. MBVs had an inhibitory effect on CXCL10 mRNA expression in human M1 macrophages (Figure 3A). This chemokine is a marker of M1 polarization, and its reduction indicates a weakening of pro-inflammatory M1 activation in M1 macrophages. sEVs also tended to reduce CXCL10 expression; however, the effect was not statistically significant (Figure 3A). An interesting effect of MBVs was observed on M2 macrophages, as these vesicles significantly increased the expression of CXCL10 mRNA relative to M2 macrophages without vesicle treatment (Figure 3A).

**Figure 4:**
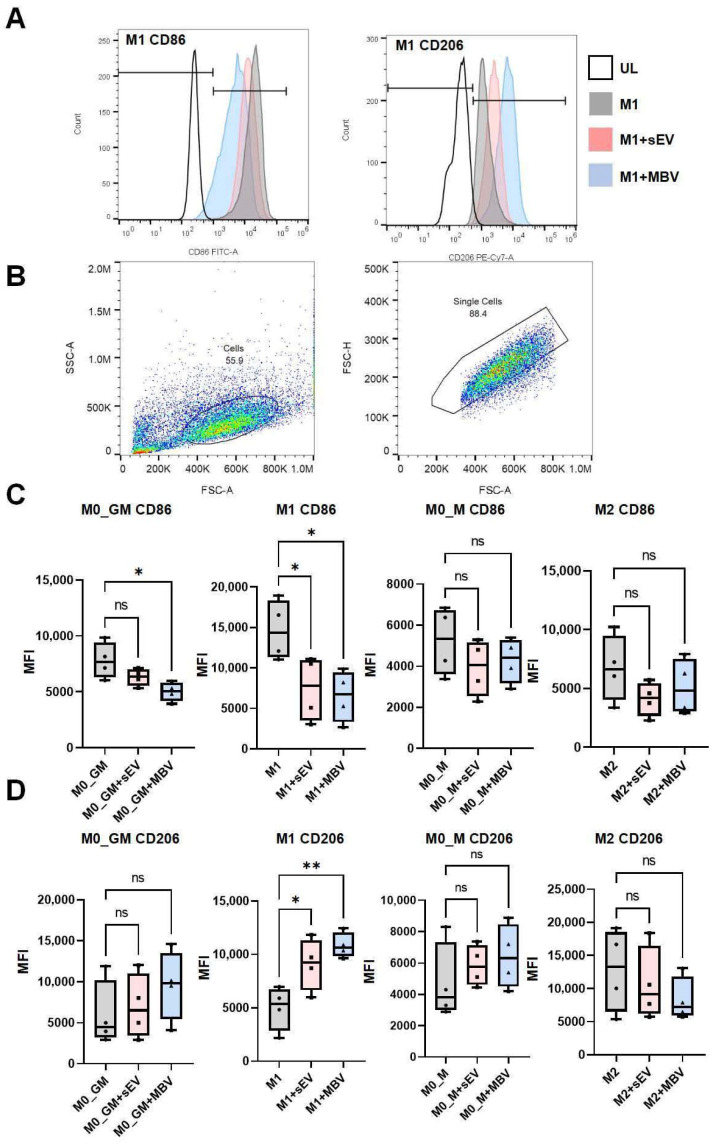
Analysis of the effects of sEV and MBV UC-MSCs on the expression of surface receptors in MDMs using flow cytometry. (**A**) Histograms showing fluorescence intensity of M1 macrophages stained with anti-CD86-FITC and anti-CD206-PE-Cy7 antibodies after incubation with UC-MSC vesicles. (**B**) A plot of forward scatter area (FSC-A) versus side scatter area (SSC-A) was used to identify cells and exclude debris. To analyze single cells, a plot of forward scatter height (FSC-H) versus FSC-A density was constructed. (**C**,**D**) Flow cytometry MFI data for M0_GM, M1, M0_M, and M2 macrophages after exposure to sEVs and MBVs derived from human UC-MSCs. (**C**) MFI for CD86-FITC, M1 polarization marker; (**D**) MFI for CD206-PE-Cy7, M2 polarization marker. The number of MDM donors is *n* = 4. Statistical analysis was performed using one-way ANOVA followed by Tukey’s multiple comparison test. This test was used for each experimental group. * *p* < 0.05; ** *p* < 0.01; ns (*p* > 0.05).

Further, we also examined the effects of sEVs and MBVs on the expression of surface receptors in MDMs of different phenotypes—M0_GM, M1, M0_M, and M2—such as CD86, a marker of M1 polarization, and CD206, a marker of M2 macrophage polarization. The expression of CD86 and CD206 was assessed at both the mRNA and protein levels using quantitative RT-PCR and flow cytometry (Figure 3B,C and Figure 4). RT-PCR analysis showed that both types of vesicles enhanced CD206 mRNA expression in M1 macrophages, with the highest expression observed in the M1 + MBV group (Figure 3C). Both vesicle types demonstrated an inhibitory effect on CD86 mRNA expression in M1 macrophages, with the greatest reduction in the M1 + MBV group (Figure 2B). MBVs significantly reduced CD86 mRNA expression in M0_GM macrophages (Figure 3B).

Flow cytometry was used to quantitatively analyze changes in CD206 and CD86 expression (Figure 4). A standard gating strategy was used to detect CD206- and CD86-positive M1-macrophages (Figure 4A,B). More detailed information on the gating strategy for detecting CD206- and CD86-positive macrophages, as well as the dot plots for all phenotypes—M0_GM, M1, M0_M, and M2—is provided in the Supplementary Materials (Figure S6). For all macrophage phenotypes, fluorescence intensity data were quantitatively presented as median fluorescence intensity (MFI) values (Figure 4C,D). The results showed a decrease in CD86 expression in M1 macrophages after exposure to sEVs and MBVs derived from UC-MSCs (Figure 4C and Figure S6). MBV UC-MSCs also exerted an inhibitory effect on CD86 mRNA expression in M0_GM macrophages (Figure 4C). Quantitative changes in CD86 and CD206 expression obtained by flow cytometry (Figure 4C,D) aligned with the qRT-PCR results for CD86 and CD206 in all types of macrophages (Figure 3B,C). The results presented in Figure 3 and Figure 4 suggest that MBV and sEV UC-MSCs reduce mRNA expression of the chemokine CXCL10, decrease the expression of surface receptors CD86, and increase CD206 in M1 macrophages, promoting an M1-to-M2-like phenotypic shift in selected markers, with less pronounced pro-inflammatory activity compared to M1 macrophages cultured without vesicles. It should also be noted that MBV UC-MSCs increased CXCL10 expression in M2 macrophages, suggesting that this vesicle type may induce a mild pro-inflammatory activation in M2 macrophages. These findings indicate that MBVs enhance the polarization of M1 macrophages toward the M2 direction, while the induced phenotype differs from M2 macrophages polarized with IL-4 (Figure 3 and Figure 4).

### 3.3. Modulation of JAK/STAT1 Components in M1 Macrophages Treated with EV UC-MSCs

**Figure 5:**
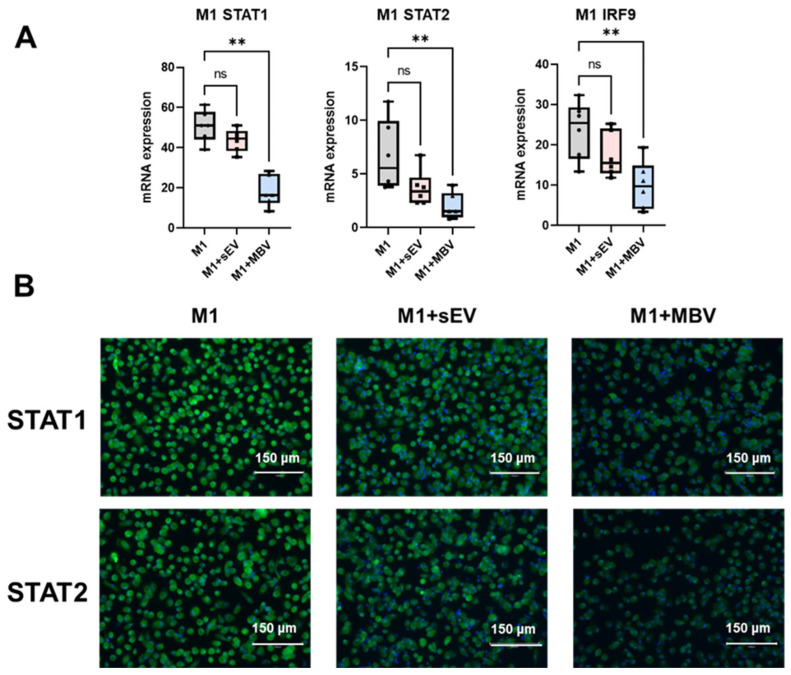
Modulation of JAK/STAT1 signaling pathway components in MDM M1 phenotype after incubation with sEVs and MBVs obtained from UC-MSCs. (**A**) qRT-PCR for STAT1, STAT2, IRF9 in M1 macrophages. (**B**) Immunocytochemical staining of STAT1, STAT2 protein expression in M1 macrophages after incubation with EV UC-MSCs. Anti-STAT1 and anti-STAT2 antibodies with anti-rabbit secondary AB labeled by Alexa Fluor 488 (green) were used; Hoechst (blue) stains the nuclei. Magnification ×20. Scale bar 150 µm. The number of MDM donors is *n* = 6. The Kruskal–Wallis nonparametric test followed by Dunn’s multiple comparison post-test was used for statistical analysis. This test was used for each experimental group. ** *p* < 0.01; ns (*p* > 0.05).

The potential mechanism underlying the anti-inflammatory properties of MBV and sEV UC-MSCs was studied. RT-qPCR analysis revealed that MBVs, to a greater extent than sEVs, reduced the expression of STAT1, STAT2, and IRF9 mRNA signaling molecules in M1 macrophages (Figure 5A). No significant changes in the expression of these molecule mRNA were observed in other MDM phenotypes; therefore, we present data only for M1 macrophages, as these signaling molecules are components of the JAK/STAT1 pathway involved in the pro-inflammatory activation of M1 macrophages. This pathway is activated by M1 polarization inducers such as IFN-γ and IFN-α.

We also performed immunocytochemical staining of M1 macrophages after exposure to EV UC-MSCs to assess the expression levels of STAT1 and STAT2 proteins (Figure 5B). The decomposition of fluorescent microscopy images is presented in Figure S7. The results of immunocytochemical staining demonstrate high fluorescence in the M1 group, indicating a high expression level of STAT1 and STAT2 proteins (Figure 5B). Exposure of M1 macrophages to sEV UC-MSCs led to a slight decrease in staining intensity, which indicates a weak effect of these vesicles on the expression of STAT1 and STAT2 proteins (Figure 5B). After exposure to MBVs, a significant decrease in staining intensity was observed, indicating a more pronounced inhibitory effect of MBVs on STAT1 and STAT2 proteins, which are components of the JAK/STAT1 signaling pathway (Figure 5B). Two types of EVs differed in their effects on the expression of mRNA of JAK/STAT1 signaling pathway molecules, as MBVs more strongly than sEVs reduced STAT1, STAT2, and IRF9 expression in M1 macrophages (Figure 5). A more pronounced decrease in STAT1, STAT2, and IRF9 in M1 macrophages after exposure to MBVs may partially explain their more pronounced effect compared to sEVs on the secretory activity and the expression of surface proteins CD86 and CD206 in the MDM M1 phenotype.

### 3.4. Analysis of the Effects of sEV and MBV UC-MSCs on Macrophage Reactive Oxygen Species Production

**Figure 6:**
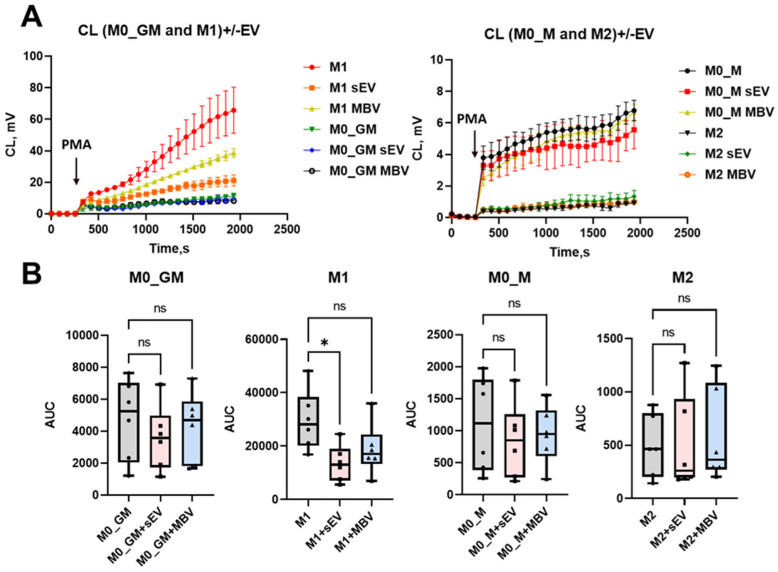
Modulation of ROS production by MDMs under the influence of sEV and MBV UC-MSCs. (**A**) Kinetic curves of luminol-dependent CL for PMA-activated M0_GM, M1, M0_M, and M2 macrophages in control and after incubation with vesicles. Arrow indicates addition of 100 ng/mL PMA; (**B**) Quantitative analysis of luminol-dependent CL results (box plot), where the *x*-axis represents experimental groups, and the *y*-axis shows the area under the CL curve (AUC). The number of MDM donors is *n* = 6. Statistical analysis was performed using the Kruskal–Wallis test with Dunn’s post hoc test. This test was used for each experimental group. * *p* < 0.05; ns (*p* > 0.05).

The ability of macrophages to generate ROS is one of the key functions of these phagocytes, as the produced free radicals, together with proteases, participate in the destruction of pathogenic microorganisms. In this study, it was found that M1 macrophages exhibited the highest radical-generating activity after activation with 100 ng/mL PMA as recorded by chemiluminescence (CL), while other macrophage phenotypes showed lower CL. The descending order of activity was M0_GM, M0_M, and M2 (Figure 6A,B). Exposure to UC-MSC-derived vesicles resulted in reduced luminol-dependent CL in M1 macrophages, manifested as a decrease in the area under the kinetic curve (AUC). The effect was most pronounced for sEVs and reached statistical significance, while MBVs caused only a downward trend (Figure 6B). In M0_GM and M0_M macrophages, a moderate decrease in ROS production was also observed following exposure to both sEVs and MBVs, although this decrease did not reach statistical significance. For M2 macrophages, UC-MSC vesicles did not exhibit any pro-oxidant activity (Figure 6B).

**Figure 7:**
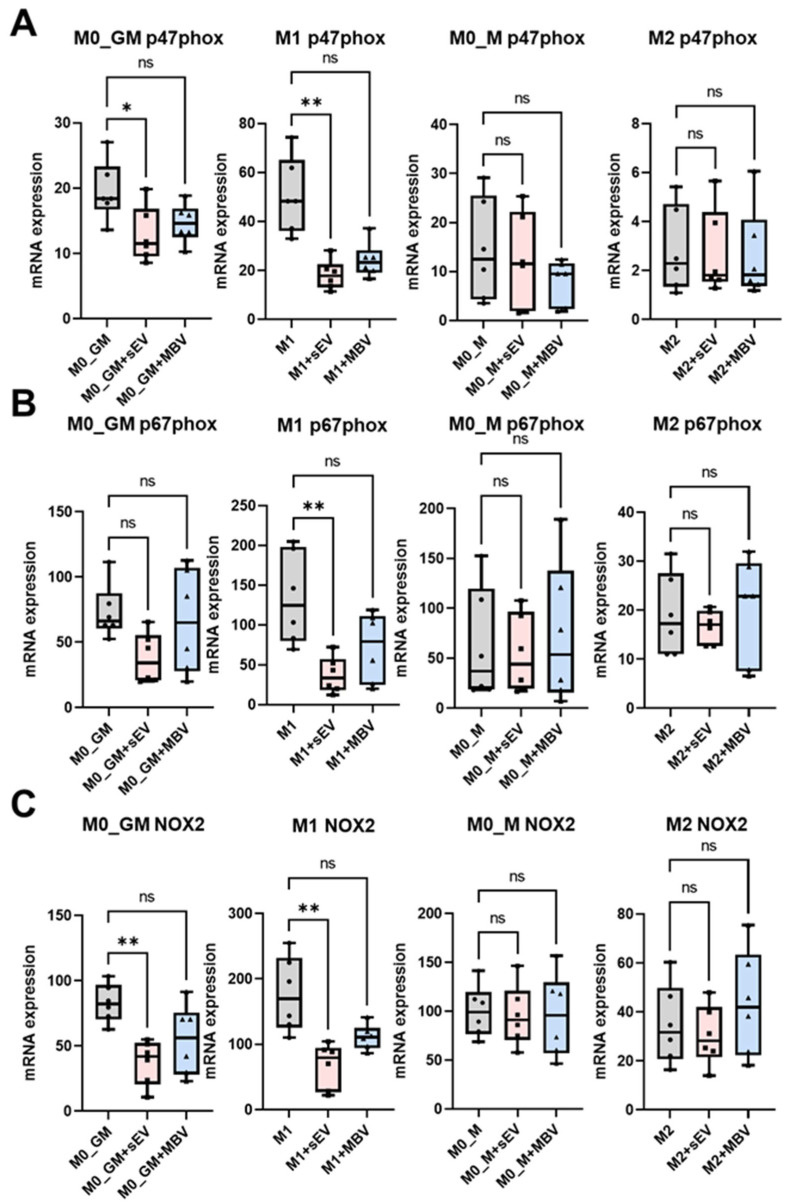
Modulation of mRNA expression of NADPH oxidase 2 and its subunits NOX-2, p47phox, and p67phox in MDM under the influence of MBVs and sEVs derived from UC-MSCs. Evaluation of mRNA expression by RT-qPCR: (**A**) p47phox; (**B**) p67phox; (**C**) NOX-2. The number of MDM donors is *n* = 6. For statistical analysis, the Kruskal–Wallis test with Dunn’s post hoc test was used. This test was used for each experimental group. * *p* < 0.05; ** *p* < 0.01; ns (*p* > 0.05).

To elucidate the mechanisms underlying the effects of UC-MSC-derived vesicles on the modulation of ROS production by M1 macrophages, RT-qPCR analysis of the expression of mRNA for NADPH oxidase 2 subunits (CYBB, encoding the β-subunit of the NOX2/gp91^phox complex), p47phox, and p67phox was performed in M0_GM, M1, M0_M, and M2 macrophages (Figure 7A–C). RT-qPCR analysis demonstrated that sEVs, more effectively than MBV UC-MSCs, reduced the expression levels of NOX2, p47phox, and p67phox in pro-inflammatory M1 macrophages, showing pronounced statistically significant effect (Figure 7A–C). sEV UC-MSCs also influenced NOX2 and p47phox expression in M0_GM macrophages, significantly decreasing the mRNA expression of these proteins (Figure 7A,C). A decrease in p67phox mRNA expression in M0_GM macrophages was observed after cultivation with sEVs, but it did not reach statistical significance (Figure 7B). sEVs and MBVs derived from UC-MSCs did not significantly affect ROS production or mRNA expression of NOX2, p47phox, and p67phox in M0_M and M2 macrophages (Figure 6 and Figure 7). The obtained results demonstrate that sEVs, to a greater extent than MBV UC-MSCs, modulate radical-generating activity in pro-inflammatory M1 macrophages, attenuating ROS production and the expression of genes associated with NADPH oxidase 2, responsible for superoxide anion generation.

### 3.5. Analysis of the Effects of sEV and MBV UC-MSCs on Macrophage Phagocytic Activity

**Figure 8:**
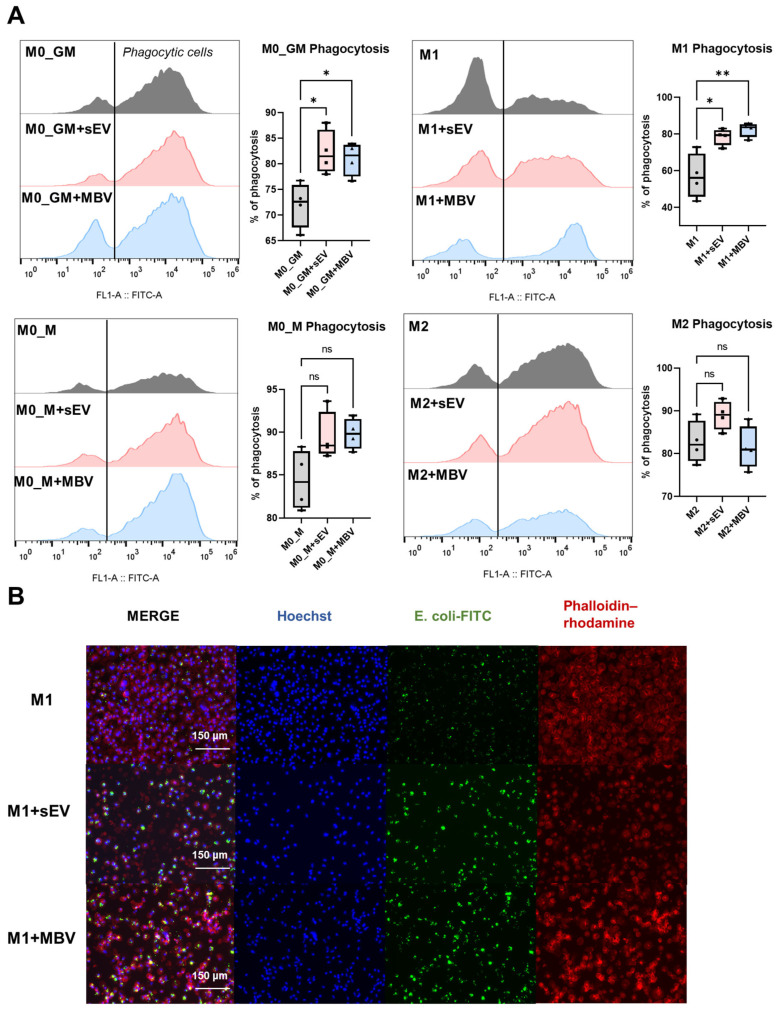
Modulation of the phagocytic activity of MDMs with different phenotypes (M0_GM, M1, M0_M, M2) by sEVs and MBVs derived from UC-MSCs. (**A**) Histograms of FITC fluorescence distribution after incubation of macrophages with *E. coli*-FITC obtained by flow cytometry. The histograms show the percentages of macrophages within the total population that phagocytosed FITC-labeled bacteria (located to the right of the demarcation line). Alongside the histograms, graphs representing the quantitative analysis of the percentage of M0_GM, M1, M0_M, and M2 macrophages phagocytosing *E. coli*-FITC after exposure to EV-UC-MSCs are shown. (**B**) Fluorescence microscopy of fixed human M1 macrophages after incubation with *E. coli*-FITC bioparticles at a 10:1 ratio (20×). Green fluorescence corresponds to FITC-labeled *E. coli* bioparticles; red indicates the actin cytoskeleton stained with phalloidin–rhodamine; blue corresponds to Hoechst, a nuclear dye. The number of MDM donors is *n* = 4. Statistical analysis was performed using ANOVA with Tukey’s post hoc test. This test was used for each experimental group. * *p* < 0.05; ** *p* < 0.01; ns—*p* > 0.05.

Flow cytometry was used to quantitatively analyze the effect of EC-MSC EVs on the phagocytosis of E-coli labeled with FITC by MDMs (Figure 8A). The black vertical line on the histograms separates phagocytic cells with a high MFI-FITC value from non-phagocytic cells with a low MFI-FITC value. The phagocytic capacity of the cells was calculated as the percentage of cells phagocytosing *E. coli*-FITC relative to the total macrophage population in the analysis, which was taken as 100%. More detailed information on the gating strategy used to detect CD206- and CD86-positive macrophages, as well as the dot plots for all phenotypes—M0_GM, M1, M0_M, and M2—is provided in the Supplementary Materials (Figure S8). During the study of the phagocytic activity of macrophages with different phenotypes, it was found that the ability of macrophages to phagocytose *E. coli*-FITC bacteria was enhanced when culturing with M-CSF and was more associated with M0_M and M2 phenotypes. Macrophages derived from human monocytes showed the lowest phagocytic ability when polarized toward M1 and M0_GM phenotypes, and the highest in M2 and M0_M phenotypes, respectively (Figure 8A). sEVs and MBVs derived from human UC-MSCs enhanced phagocytosis in M0_GM and M1 macrophages (Figure 8A). For M0_M and M2 macrophages, a tendency toward increased phagocytosis of *E. coli*-FITC bacteria was observed after incubation with sEVs, although the effects were not statistically significant, while MBVs slightly inhibited this activity in M2 macrophages (Figure 8A). Microphotographs of *E. coli*-FITC phagocytosis in M1 macrophages showed a tendency toward increased phagocytosis both in intensity and in the number of cells that had phagocytosed fluorescent particles (Figure 8B) which was also observed in M0_GM, but in M0_M and M2 phenotypes (Figure S9). These results suggest that MBV and sEV UC-MSCs induce changes in the functional activity of M0_GM and M1 macrophages, making their phagocytic activity comparable to that of the anti-inflammatory phenotypes M2 and M0_M cultured with M-CSF.

## 4. Discussion

Macrophages are a key population of mononuclear phagocytes present in nearly all human organs. They play essential roles in protecting against pathogens, regulating tissue homeostasis, participating in regeneration and contributing to the development of various diseases [48,49,50]. Control of the balance between pro-inflammatory M1 and anti-inflammatory regenerative M2 phenotypes is considered a promising therapeutic strategy. Shifting the polarization toward M2-like macrophages can promote inflammation resolution, reduce destructive processes, and stimulate regeneration [51,52].The use of vesicles derived from MSCs from various tissues represents a promising therapeutic direction for treating degenerative and metabolic diseases and wound healing due to multiple mechanisms of actions, including immunomodulatory and anti-inflammatory effects [53,54,55]. For example, vesicles derived from UC-MSCs attenuate the inflammation in rats with burn-induced injury by targeting miR-181c, effectively suppressing the Toll-like receptor 4 (TLR4) signaling pathway in M1 macrophages activated to a pro-inflammatory state by LPS [56]. UC-MSC-derived vesicles also exhibit a significant anti-inflammatory effect, alleviating hyperglycemia and multiorgan inflammation associated with type 1 diabetes mellitus. This was achieved by influencing macrophage polarization through the delivery of insulin and superoxide dismutase-1, thereby reducing the secretion of pro-inflammatory factors such as IL-6, TNF-α, and CCL-2 [57]. These findings highlight the remarkable therapeutic potential of MSC-derived vesicles in promoting regenerative processes across various diseases by modulating macrophage plasticity.

In recent years, there has been a growing interest on MBVs derived from the extracellular matrix or cell cultures. They exhibit significant regenerative and anti-inflammatory potential in rheumatoid arthritis and osteoarthritis [34,58]. MBVs are obtained through decellularization of the ECMs from soft tissue mucosa, while monolayer cell cultures can also serve as source for these vesicles. For instance, MBVs have been successfully isolated from mouse fibroblast NIH-3T3 cell lines and MSC spheroids [34,42,59]. MBVs are rich in microRNA, containing over 200 miRNA species. The mechanisms by which MBVs influence the behavior of neighboring cells and macrophage activation remain not fully understood [60]. It has been established that three miRNAs—miR-125b-5p, miR-143-3p, and miR-145-5p—contained in MBVs derived from bladder and small intestinal extracellular matrices promote macrophage polarization toward an anti-inflammatory M2 state [43]. A comparative study of MBVs obtained from MSC cultured as 2D monolayers versus 3D spheroids demonstrated differential activity toward THP-1 macrophages: 2D MBVs generally suppressed the pro-inflammatory cytokine IL-12β, while 3D MBV enhanced anti-inflammatory cytokine IL-10 [59]. MBVs derived from human UC-MSC exhibit strong anti-inflammatory properties on human monocyte-derived M1 macrophages [34]. In our previous study, both MBVs and exosomes attenuate inflammatory and degenerative processes in the knee joints of rats with osteoarthritis, reducing levels of inflammatory markers (TNF-α, iNOS) and increasing Arg-1 expression in macrophages and synovial fibroblasts, indicating a stronger anti-inflammatory effect of exosomes [34]. These results demonstrate heterogeneous effects of MBVs and sEVs derived from MSC secretome. The findings from the above-mentioned studies reveal differences between the effects of MSC-derived MBVs and sEVs on macrophages in vitro and in vivo under various culture conditions.

Our study demonstrated that UC-MSC-derived MBVs promote a more pronounced decrease in CD86 receptor expression and mRNA CXCL10, characteristic of M1 macrophage polarization, compared to sEVs, along with a significant increase in CD206 expression in M1 macrophages after exposure to UC-MSC vesicles. These findings are consistent with our previously obtained results: MBVs more effectively reduce the expression and secretion of inflammatory cytokines IL-6 and TNF-α compared to exosomes from the same cellular source [34]. Our results indicate the modulation of polarization markers in M1 macrophages under the influence of UC-MSC vesicles, resulting in the shift in M1 macrophage to M2-like phenotype in selected markers.

Under the influence of diverse chemical signals, macrophages can switch their phenotype across a wide spectrum between M1 and M2 macrophage phenotypes, which represent the conditional extremes of the continuum of macrophage phenotypic variability [61]. Macrophage polarization is regulated by several signaling pathways, including JAK/STAT, NF-κB, PI3K/AKT, Notch, MAPK, and Wnt [62,63,64,65]. Depending on the specific signaling molecule, the components of these pathways can promote either pro-inflammatory M1 or anti-inflammatory M2 macrophage polarization [66]. For instance, the activation of STAT1, STAT2, IRF9, and NF-κB p65 is characteristic of M1 macrophages, whereas STAT3, STAT6, and NF-κB p50 enhance M2 polarization [67]. Of particular importance is the JAK/STAT pathway, which mediates responses to more than 70 cytokines and is crucial for regulation immune homeostasis [68]. This signaling pathway plays a central role in macrophage phenotypic plasticity, as its dysregulation can contribute to immune disorders, including autoimmune diseases, cancer, and immunodeficiency [68]. The results of this study show that MBVs derived from UC-MSCs decreased the mRNA expression of STAT1, STAT2, and IRF9 mRNA in M1 macrophages. Using immunocytochemical staining, we also demonstrated a decrease in the expression of STAT1 and STAT2 proteins in M1 macrophages after exposure to MBV UC-MSCs, which is consistent with the results obtained using RT-qPCR. sEVs did not exert a significant inhibitory effect on the expression of these signaling molecules, although a decreasing trend in mRNA expression of these pathway components was observed. These data suggest that the more pronounced changes in CXCL10, IL-6 and TNF-α, CD86, and CD206 expression in M1 macrophages under the influence of MBVs may be associated with reduced activation of the JAK/STAT1/STAT2 signaling pathway. In the field examining the relationship between specific cargos contained in sEVs and MBVs, several miRNAs have been identified that modulate JAK/STAT signaling pathways in macrophages, for example, miR-26a and miR-21, which through different mechanisms enhance STAT3 expression, thereby promoting anti-inflammatory M2-like polarization [69,70]. miR-21 targets PTEN, which leads to increased phosphorylation of both AKT and STAT3, while miR-26a reduces TLR3 expression, attenuating NF-κB activation and consequently promoting STAT3 phosphorylation and M2 polarization [71,72]. In the case of MBVs obtained from MSCs, there is insufficient evidence to support their involvement in regulating STAT1/STAT2/IRF9 expression in M1 macrophages, as well as the contribution of specific miRNAs to this process. Therefore, this question requires further investigation.

The radical-generating activity and ROS formation by macrophages are essential for both immune defense and physiological functions [73]. ROS play important roles in monocyte differentiation, intracellular signaling, gene regulation, and cell death induction [74]. ROS also play an important role in regulating physiological processes in the body, and an imbalance between the pro-oxidant activity of macrophages and the antioxidant defense system can lead to excessive ROS generation and subsequent tissue and organ damage [7]. One of the main sources of ROS in macrophages is NADPH oxidase, which catalyzes the formation of superoxide anion (O_2_•^−^) and is mainly represented by the NOX2 isoform [75]. Elevated ROS production by M1 macrophages ensures antibacterial and antitumor defense [14]. However, the dysregulation of macrophage phenotype may lead to excessive ROS production, aggravating pathological processes. For example, in traumatic brain injury, a shift toward the M1 microglial profile is accompanied by increased NOX2 expression and oxidative damage to cortical tissue [76,77]. Reduced NADPH oxidase activity and increased M2 macrophage proportion can mitigate destructive inflammation, preserving tissue integrity [76]. In this study, MBVs and sEVs derived from UC-MSCs reduced ROS (O_2_•^−^/H_2_O_2_) production by M1 macrophages, as measured by luminol-dependent chemiluminescence. The inhibitory effect on ROS production was statistically significant for sEVs but not for MBVs. Additionally, RT-qPCR analysis revealed that both vesicle types suppressed NOX2 (CYBB), p47phox (NCF1), and p67phox (NCF2) mRNA expression in M1 macrophages, decreasing NADPH oxidase 2 subunit transcription. The effect of sEVs on p47phox and p67phox mRNA expression was more pronounced than that of MBVs in M1 macrophages. To date, the precise mechanism by which sEVs and MBVs derived from UC-MSCs modulate ROS production in MDMs remains to be clarified. Future studies should determine the specific cargos of these EVs responsible for modulating NOX2 expression and the reasons underlying the differential suppression of NOX2, p47phox, and p67phox subunits in M1 macrophages, as the exact mechanisms and specific components of sEV cargos—primarily miRNAs and proteins are currently unknown. Other studies have demonstrated an association between miR-100-5p derived from human UC-MSC sEVs and decreased ROS production and NOX4 expression in chondrocytes in an osteoarthritis model, as well as in dopaminergic neurons in a Parkinson’s disease model [78,79].

Phagocytosis is one of the key macrophage functions, along with the production of ROS, enabling the engulfment and destruction of pathogenic microorganisms and cell debris. During this process, phagosomes are formed and fuse with proteolytic enzymes that degrade engulfed material [80]. Phagocytic activity is characteristic of both M1 and M2 macrophages, as demonstrated in RAW 264.7 macrophages [81]. However, some studies have reported reduced phagocytic activity following polarization toward the M2 phenotype. In one study, IL-4-induced alternative activation of mouse peritoneal macrophages significantly reduced phagocytosis of N. meningitidis while enhancing cathepsin production and increasing phagosomal acidity and proteolytic activity [82]. In the case of Porphyromonas gingivalis, the bacterium that causes periodontitis, M1 and, to a greater extent, M2 macrophages of the RAW 264.7 line, had a stronger phagocytic activity compared to unprimed macrophages [81]. Phagocytosis of these bacteria by M1 macrophages led to a respiratory explosion and pro-inflammatory activation, while, in unprimed and M2 macrophages, *P. gingivalis* persisted for 24 h [81]. The studies on human macrophages have revealed that the phagocytosis rate of *E. coli* is higher in M2 macrophages compared to M1 macrophages [14,83]. However, it is worth noting that, in some studies, the opposite picture was observed in the case of phagocytosis by macrophages of carboxylated polystyrene nanoparticles. M1 macrophages showed greater phagocytic activity compared to M2 macrophages obtained from the bone marrow of mice. In the case of the modification of the surface of CD47 nanoparticles, the level of phagocytosis in M1 macrophages decreased compared to M2 macrophages [84]. Phagocytic activity may depend on the pathogen used in the experiment, which complicates the generalization of results [43]. In a study evaluating MBVs isolated from bladder and small intestinal submucosa ECMs, bone-marrow-derived M1 macrophages (IFN-γ + LPS) showed the highest phagocytic activity compared to M0 and M2 macrophages [43]. Exposure to MBVs of both types enhanced *E. coli*-FITC-phagocytosis by M0 macrophages [43]. In the present study, macrophage phagocytic activity increased from M1 and M0_GM to M2 and M0_M, reaching its maximum in M2 and M0_M. MBVs and sEVs derived from UC-MSCs enhanced phagocytic activity in M1 and M0_GM macrophages (measured via flow cytometry using FITC-labeled *E. coli*). The enhancement of phagocytosis in M1 macrophages under the influence of MBVs was more pronounced than that induced by sEVs and approached the level observed in M2 macrophages which is an anti-inflammatory phenotype. Based on the distinct properties of different UC-MSC-derived EV subpopulations in the regulation of human macrophage functional activity in vitro, the combined use of two EV populations (sEVs and MBVs) may provide the most effective pro-regenerative outcome in therapies for various inflammatory diseases in vivo. Specifically, sEVs are likely to more effectively inhibit ROS production compared to MBVs, whereas MBVs may more efficiently modulate the activation of pro-inflammatory M1 macrophages toward a phenotype characterized by reduced secretion of pro-inflammatory cytokines and chemokines and enhanced phagocytic activity.

The results of this study were obtained using MDMs in an in vitro setting. Several limitations should be noted. For the analysis of M1/M2 macrophage polarization markers, we used a relatively narrow marker panel: CXCL10 and CD86 for M1 macrophages, and CD206 for M2 macrophages. Although in our previous work we also assessed the effects of these EVs on TNF-α and IL-6 expression [34], markers associated with M2 polarization such as CD163, IL-10, and TGF-β were not evaluated in this study. Another important limitation of this study is the normalization of sEVs and MBVs derived from UC-MSCs based on protein content rather than particle-number normalization. When differential ultracentrifugation is applied without additional protein-depletion or purification steps, residual protein contamination may occur, which may not accurately reflect the true protein content of the EV fraction. Therefore, future studies would benefit from implementing particle-based normalization approaches, such as nanoparticle tracking analysis, and/or from employing EV isolation strategies that reduce protein contamination. It should also be noted that the assays used to evaluate the effects of sEVs and MBVs on macrophage phagocytosis of *E. coli* by flow cytometry and PMA-induced ROS production measured by chemiluminescence may not fully reflect conditions of sterile inflammation or the tumor microenvironment. Therefore, caution should be exercised when interpreting these findings in other inflammatory contexts. Another limitation of this study is the insufficient analysis of the effects of specific cargos, such as miRNAs and proteins, within both sEVs and MBVs derived from UC-MSCs on the modulation of STAT1/STAT2/IRF9 signaling under MBV treatment and NOX2 regulation under sEV treatment. Future studies will include more detailed transcriptomic profiling of both EV types, as well as sequencing of macrophages themselves, in order to establish links between differences in EV composition, regulatory RNA content, and the modulation of signaling pathways affecting macrophage polarization and functional activity. Future studies should focus on elucidating the qualitative and quantitative compositional differences between various UC-MSC-derived EV populations to identify possible mechanistic factors underlying the distinct immunomodulatory effects observed between the two subpopulations (sEVs and MBVs). Another promising direction involves engineering the development of modified sEV and MBV scaffolds capable of exerting more potent anti-inflammatory and regenerative effects in the treatment of inflammatory or degenerative diseases in vivo; however, this hypothesis requires thorough experimental validation.

## 5. Conclusions

This study expands the understanding of how different vesicle types—MBVs and sEVs—derived from UC-MSCs modulate the phenotypic plasticity and functional activity of human macrophages in vitro. Both vesicle types exhibited the most significant immunomodulatory effects on pro-inflammatory M1 macrophages. MBVs more strongly influenced CXCL10, CD86, and CD206 expression compared to sEVs, possibly due to a more pronounced decrease in JAK/STAT1 signaling pathway component expression associated with M1 polarization. MBVs and sEVs enhanced the phagocytic ability of M1 and M0_GM macrophages toward *E. coli*, elevating activity to a level approaching that of M2 macrophages. Differences were also observed in ROS modulation among macrophage phenotypes, with sEVs showing a stronger inhibitory effect on ROS production and mRNA expression of NOX2, p47phox, and p67phox subunits in M1 macrophages compared to MBVs. These findings provide valuable insights for researchers studying the therapeutic potential of MSC-derived vesicles for treating degenerative and inflammatory diseases.

## Data Availability

Data generated during the study and included in this article are available from the corresponding authors upon request.

## Supplementary Materials

The following supporting information can be downloaded at https://www.mdpi.com/article/10.3390/cells15020093/s1. Figure S1. A-Visualization of UC-MSCs EVs via transmission electron microscopy. B-Visualization of UC-MSCs EVs via confocal microscopy with fluorescent staining of UC-MSCs sEVs and UC-MSCs MBVs using PKH26 membrane dye. Figure S2. Visualization of UC-MSCs EVs exosomal positive and negative markers via western-blotting. Figure S3. Particle size distribution (PSD) of UC-MSCs sEVs and MBVs obtained by nanoparticle dynamic light scattering. The Y-axis shows the number of particles of a certain size (the mean percentage), the X-axis shows the particle size (d, nm). Figure S4. Phagocytosis of EVs UC-MSC labeled with PKH26 by M1 (IFN-γ + LPS) macrophages. PKH26 has red fluorescence; Hoechst, a nuclear dye, is shown in blue. Magnification ×100. Scale bar 50 µm. Figure S5. Analysis of the effect of different protein-normalized concentrations of EV-UC-MSCs on TNF-α secretion by M1 macrophages. Statistical analysis was performed using ANOVA with Tukey’s post hoc test. *n* = 4, ** *p* < 0.01; *** *p* < 0.001; ns—*p* > 0.05. Figure S6. Detection of the effects of sEVs and MBVs UC-MSCs on the expression of surface receptors in MDM macrophages using flow cytometry. Figure S7. Visualization of proteins STAT1, STAT2 expression in MDM of M1 phenotype after exposure to sEV and MBV UC-MSCs, obtained by immunocytochemical staining. Figure S8. Detection by flow cytometry of phagocytosis of *E. coli* bioparticles after exposure of MDM to sEVs and MBVs UC-MSCs. Figure S9. Visualization of phagocytosis of *E. coli* bioparticles by MDM of M0_GM, M0_M, and M2 phenotypes after exposure to sEVs and MBVs UC-MSCs obtained by fluorescence microscopy of fixed human macrophages after incubation with *E. coli* bioparticles at a 10:1 ratio.

## Abbreviations

AUC, Area under the curve; CXCL10, C-X-C motif chemokine ligand 10; CL, Chemiluminescence; CD, cluster of differentiation; ECM, extracellular matrix; EVs, extracellular vesicles; GAPDH, Glyceraldehyde-3-phosphate dehydrogenase; GM-CSF, granulocyte-macrophage colony-stimulating factor; IFN-γ, Interferon-γ; iNOS, inducible nitric oxide synthase; IL, interleukin; IRF9, interferon regulatory factor; JAK, Janus kinase; LPS, lipopolysaccharide; M-CSF, macrophage colony-stimulating factor; MBV, matrix-bound nanovesicle; MFI, median fluorescence intensity; MSCs, mesenchymal stromal cells; MDMs, monocyte-derived macrophages; miR, microRNA; NF-κB, nuclear factor kappa-light-chain-enhancer of activated B cells; NOX2, NADPH Oxidase 2; PMA, Phorbol 12-myristate 13-acetate; PBMC, peripheral blood mononuclear cell; PBS, phosphate-buffered saline; qRT-PCR, quantitative reverse transcription polymerase chain reaction; ROS, reactive oxygen species; sEVs, small extracellular vesicles; TLR4, toll-like receptor 4; TEM, transmission electron microscopy; TNF, tumor necrosis factor; UC-MSCs, umbilical cord mesenchymal stromal cells.
