# Supplementary material for: Mesenchymal Stem Cell-Derived Extracellular Vesicles Attenuate Pro-Inflammatory Macrophage Polarization: Comparison of Matrix-Bound and Small Extracellular Vesicles
Source: Cells. 2026 Jan 6;15(2):93. doi: 10.3390/cells15020093 (PMC12838643; doi:10.3390/cells15020093)
Supplement: Supplementary file 1 [file cells-15-00093-s001.zip › cells-4024284-supplementary.pdf]

Supplementary Material for

Mesenchymal Stem Cell-Derived Extracellular Vesicles Attenuate Pro-Inflammatory Macrophage Polarization: Comparison of Matrix-Bound and Small Extracellular Vesicles

Timofey O. Klyucherev [1,2], Maria D. Yurkanova [1,2], Daria P. Revokatova [1,2], Dmitriy A. Chevalier [1,2], Vsevolod V. Shishkov [1], Irina I. Vlasova [1], Nastasia V. Kosheleva [1,2] and Peter S. Timashev [1,*]

[1] Institute for Regenerative Medicine, I. M. Sechenov First Moscow State Medical University (Sechenov University), 119991 Moscow, Russia

[2] Laboratory of Clinical Smart Nanotechnologies, Institute for Regenerative Medicine, I. M. Sechenov First Moscow State Medical University (Sechenov University), 119991 Moscow, Russia

* Correspondence: timashev_p_s@staff.sechenov.ru

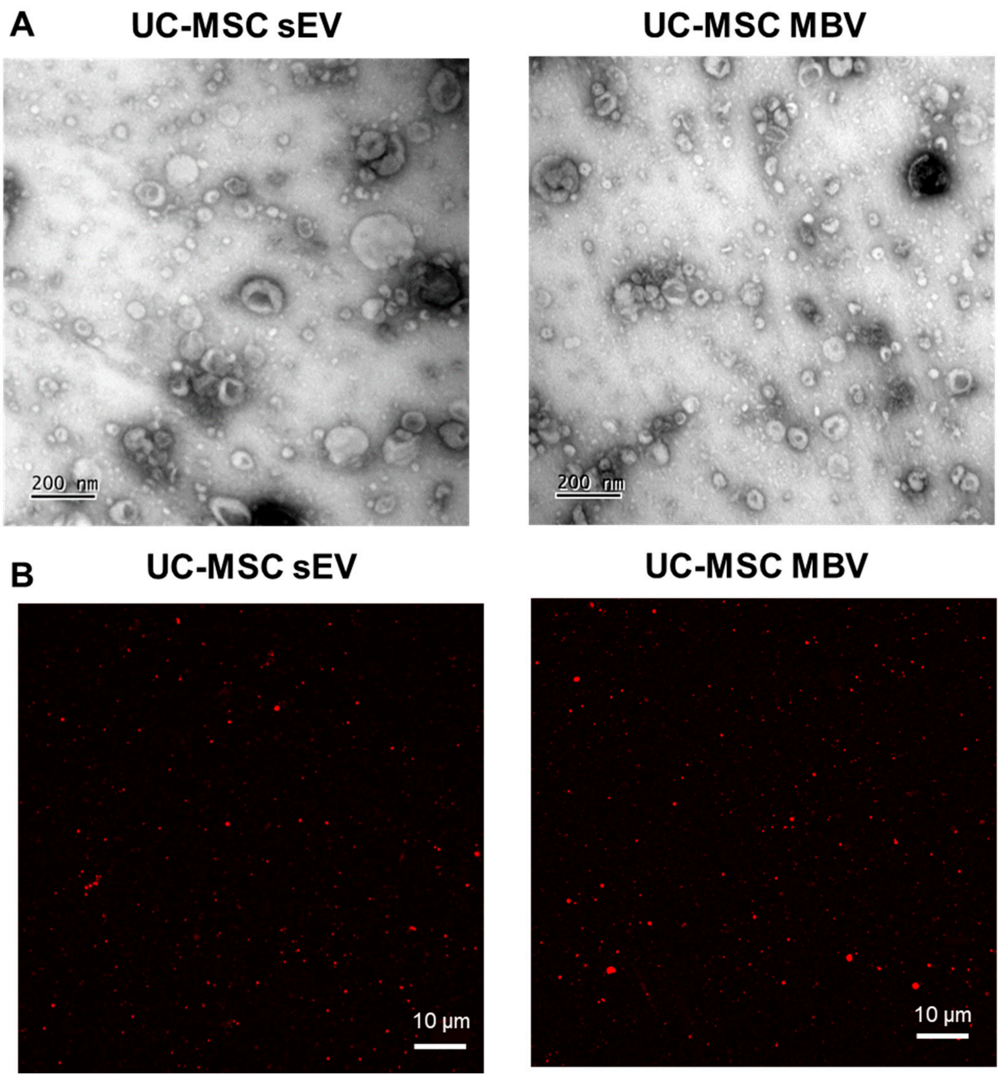

Figure S1. A - Visualization of UC-MSCs EVs via transmission electron microscopy. B - Visualization of UC-MSCs EVs via confocal microscopy with fluorescent staining of UC-MSCs sEVs and UC-MSCs MBVs using PKH26 membrane dye.

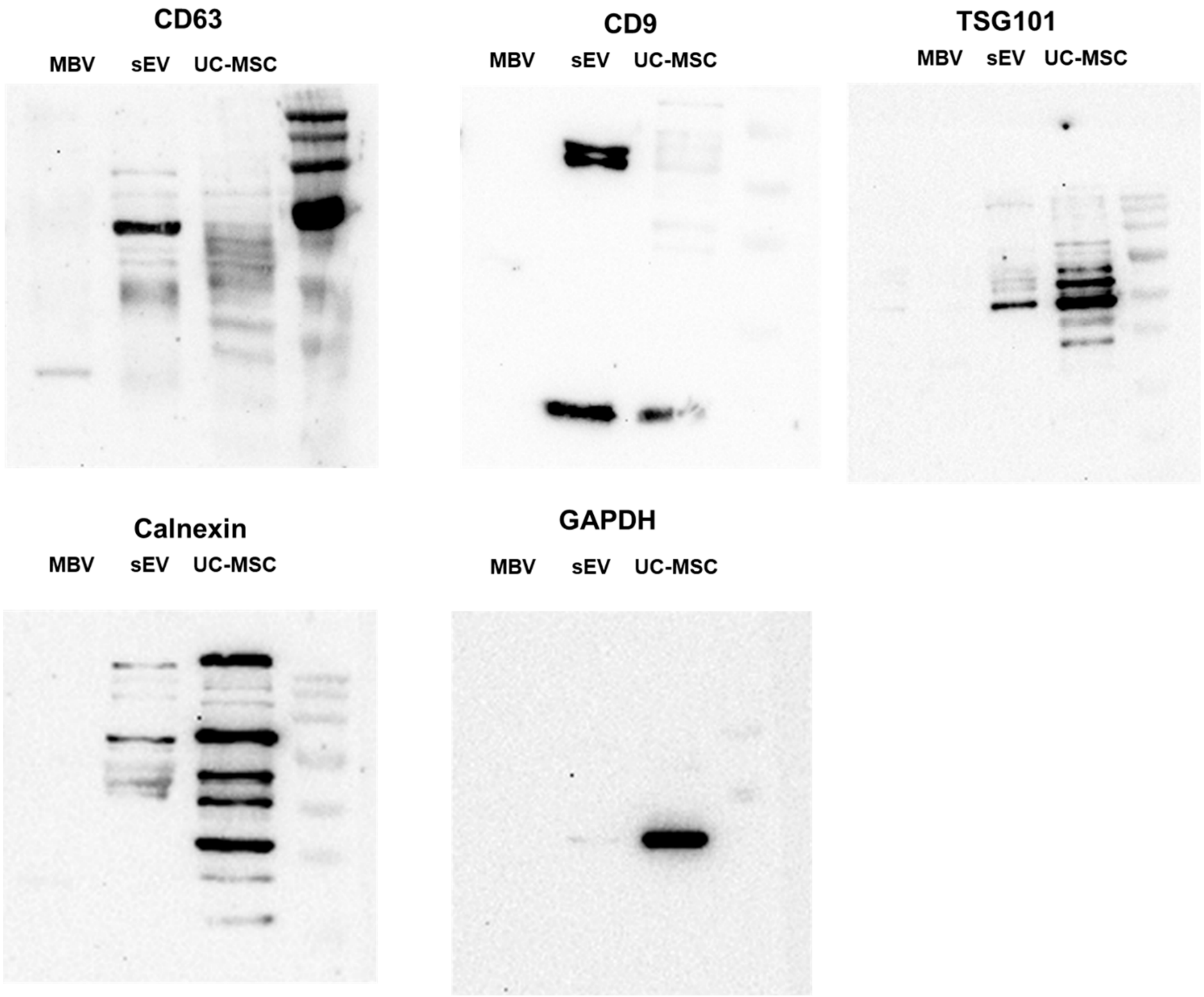

Figure S2. Visualization of UC-MSCs EVs exosomal positive and negative markers via western-blotting

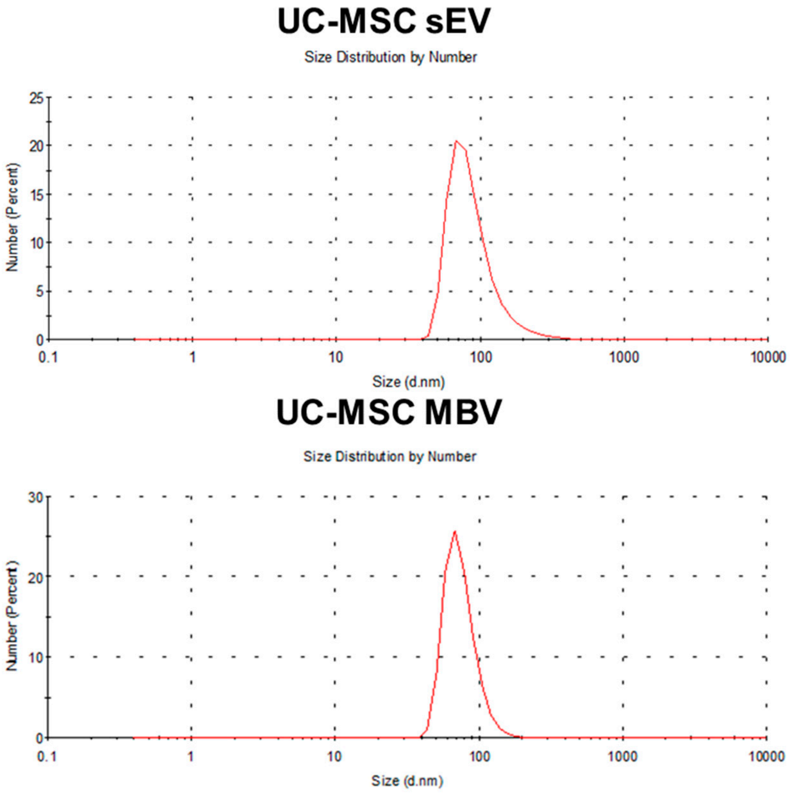

Figure S3. Particle size distribution (PSD) of UC-MSCs sEVs and MBVs obtained by nanoparticle dynamic light scattering. The Y-axis shows the number of particles of a certain size (the mean percentage), the X-axis shows the particle size (d, nm).

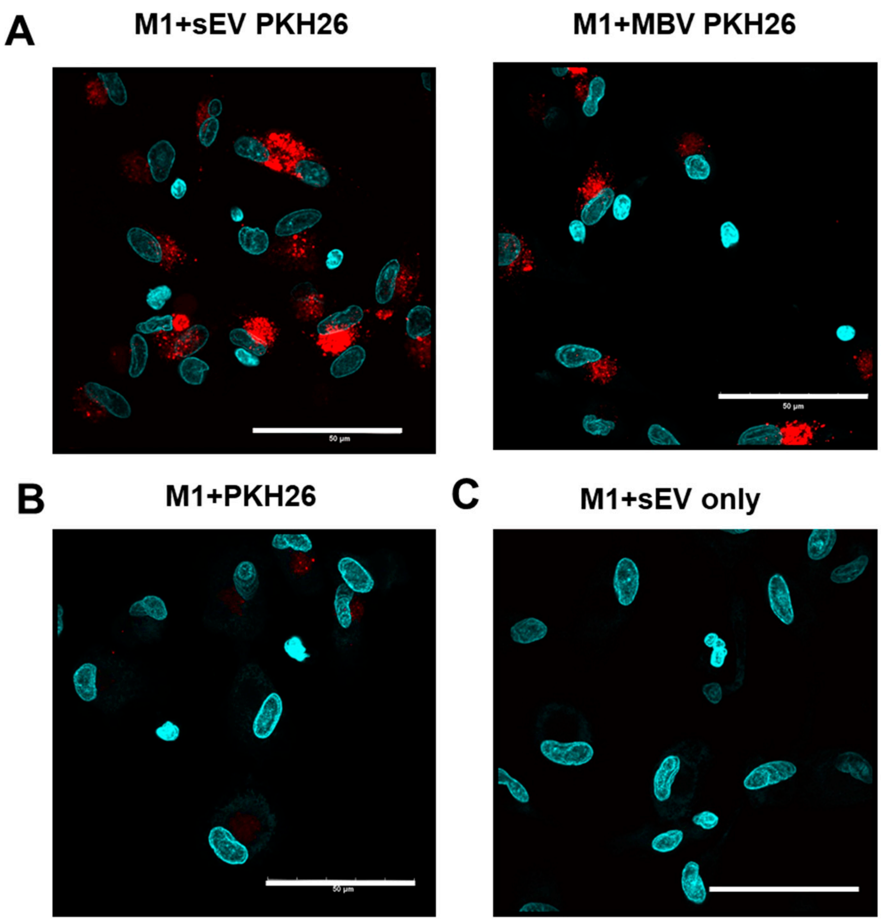

Figure S4. Phagocytosis of EVs UC-MSC labeled with PKH26 by M1 (IFN-γ+LPS) macrophages. PKH26 has red fluorescence; Hoechst, a nuclear dye, is shown in blue. Magnification ×100. Scale bar 50 µm.

The ability of macrophages to phagocytose vesicles was demonstrated for both sEVs and MBVs labeled with the membrane dye PKH26, using M1 macrophages as an example (Figure S4). For both types of vesicles labeled with PKH26, intense fluorescence of phagocytized vesicles associated with this dye was observed (Figure S4A). To assess phagocytosis of unbound PKH26 dye that remains residually present in vesicle samples, M1 macrophages were cultured with the dye precipitated without vesicles. The results showed weak phagocytosis of this dye (Figure S4B). The fluorescence intensity of the cells was significantly lower than that in M1 samples incubated with sEVs and MBVs labeled with PKH26 (Figure S4A,B). These results suggest that the PKH26-labeled vesicle samples were predominantly cleared of free dye, and that the intracellular red fluorescence is due to phagocytosis of PKH26-labeled vesicles rather than free PKH26 dye or its micelles. As an additional control, unlabeled sEVs were added to M1 macrophages; no PKH26 signal was detected in these cells (Figure S4C).

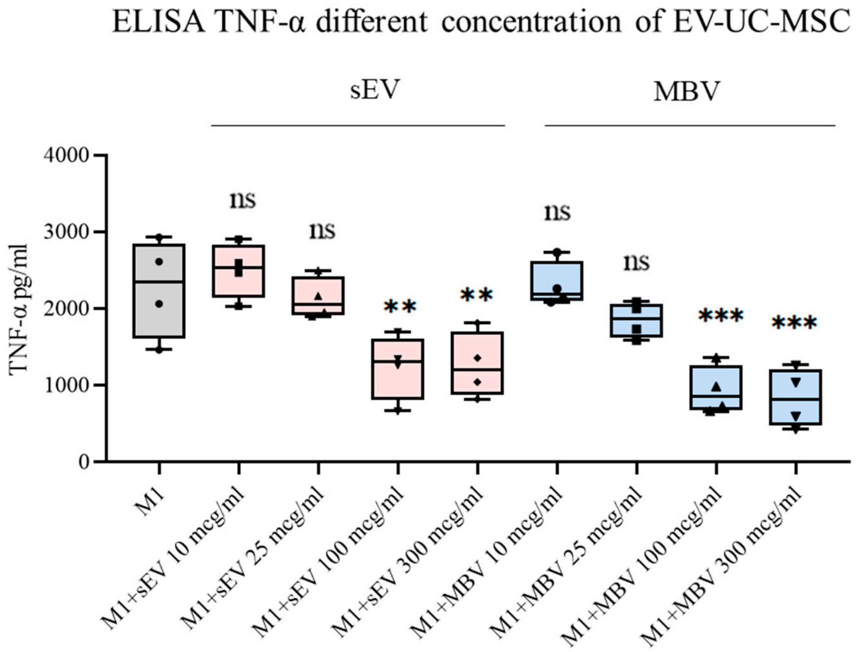

Figure S5. Analysis of the effect of different protein-normalized concentrations of EV-UC-MSCs on TNF-α secretion by M1 macrophages. Statistical analysis was performed using ANOVA with Tukey's post hoc test. n=4, **$p < 0.01$; ***$p < 0.001$; ns – $p > 0.05$.

The effect of various protein-normalized concentrations of EV-UC-MSCs on TNF-α production by M1 macrophages was assessed using ELISA. Across the series of measurements, it was found that both types of EVs significantly reduced TNF-α production in M1 macrophages at a concentration of 100 µg/ml, with no substantial differences compared to the 300 µg/ml concentration. Based on these findings, a concentration of 100 µg/ml EVs was used in the subsequent experiments.

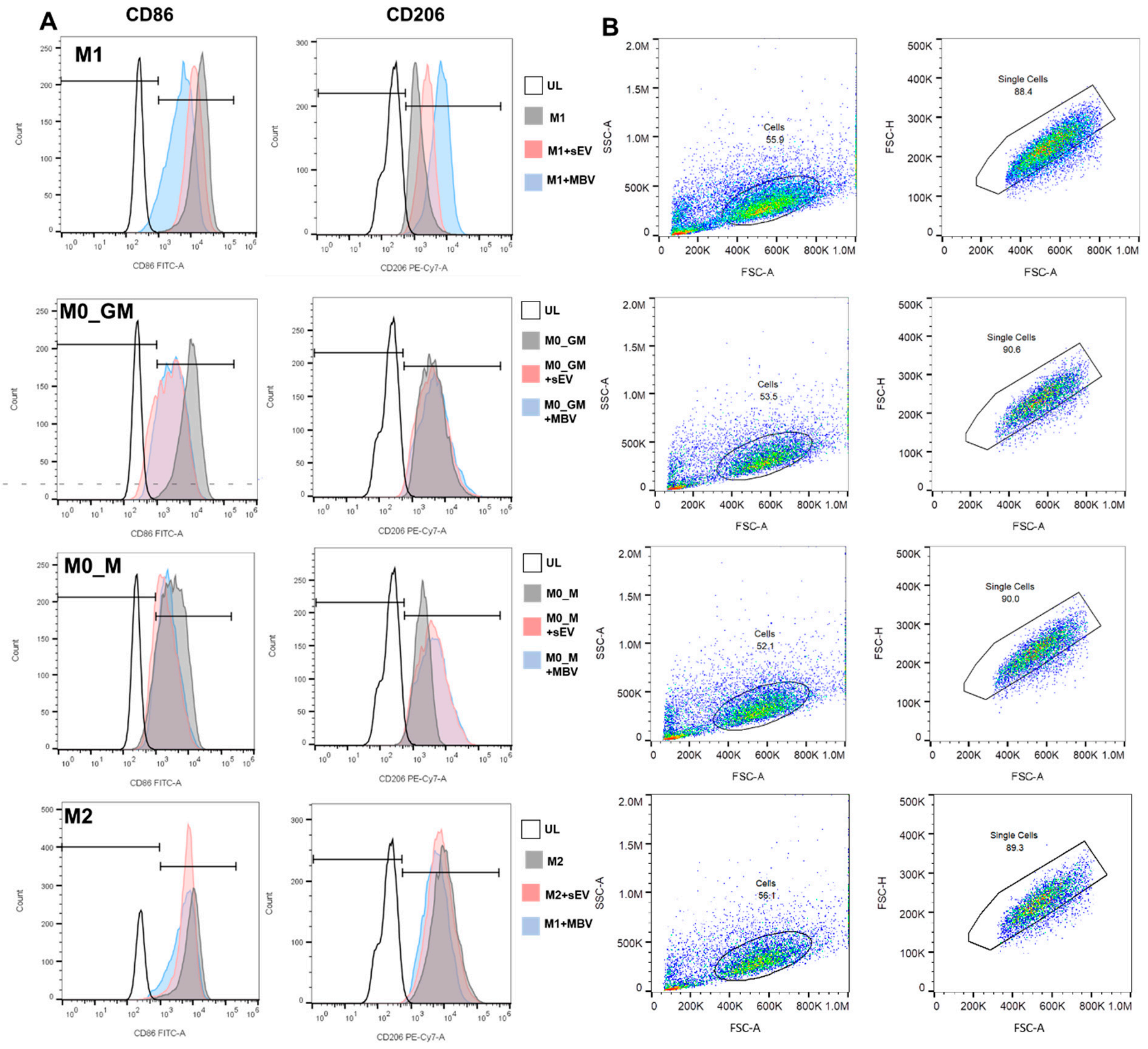

Figure S6. Detection of the effects of sEVs and MBVs UC-MSCs on the expression of surface receptors in MDM macrophages using flow cytometry. A – Histograms showing fluorescence intensity of M1, M0_GM, M0_M, and M2 macrophages after incubation with UC-MSC vesicles stained with anti-CD86-FITC and anti-CD206-PE-Cy7 antibodies. B – A plot of forward scatter area (FSC-A) versus side scatter area (SSC-A) was used to identify cells and exclude debris. To analyze single cells, a plot of forward scatter height (FSC-H) versus FSC-A density was constructed.

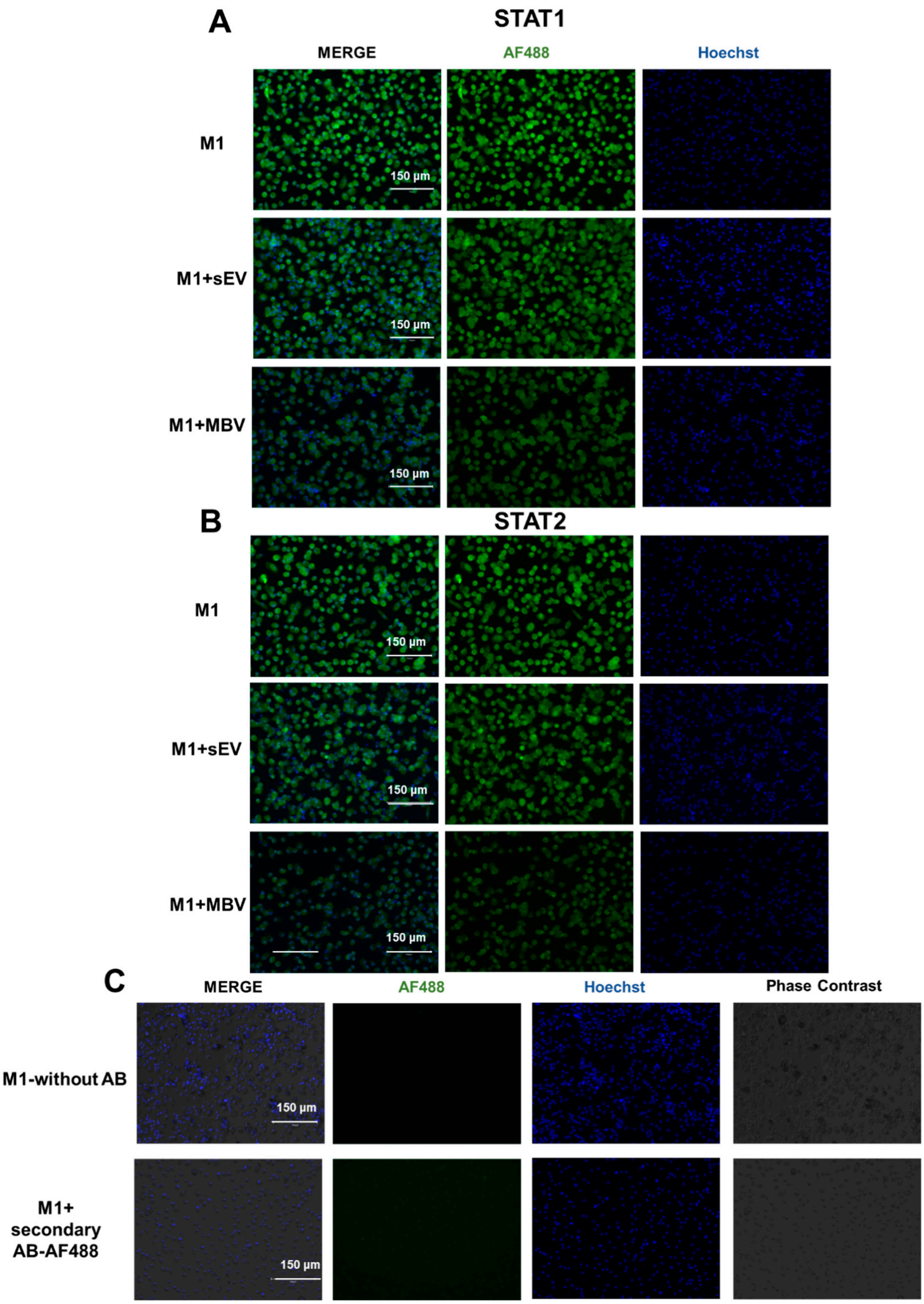

Figure S7. Visualization of proteins STAT1, STAT2 expression in MDM of M1 phenotype after exposure to sEV and MBV UC-MSCs, obtained by immunocytochemical staining. A – M1+EV macrophages labelled with anti-STAT1 antibodies (AB). Anti-rabbit secondary AB labelled by Alexa Fluor 488 were used. B – M1+EV macrophages labelled with anti-STAT2 AB and secondary AB-AlexaFluor. C – Negative control of M1 macrophages incubated without AB and M1 macrophages incubated with anti-rabbit secondary AB;

Hoechst (blue) stains the nuclei. The phase contrast (gray) shows the morphology of the cells. Magnification ×20. Scale bar 150 µm.

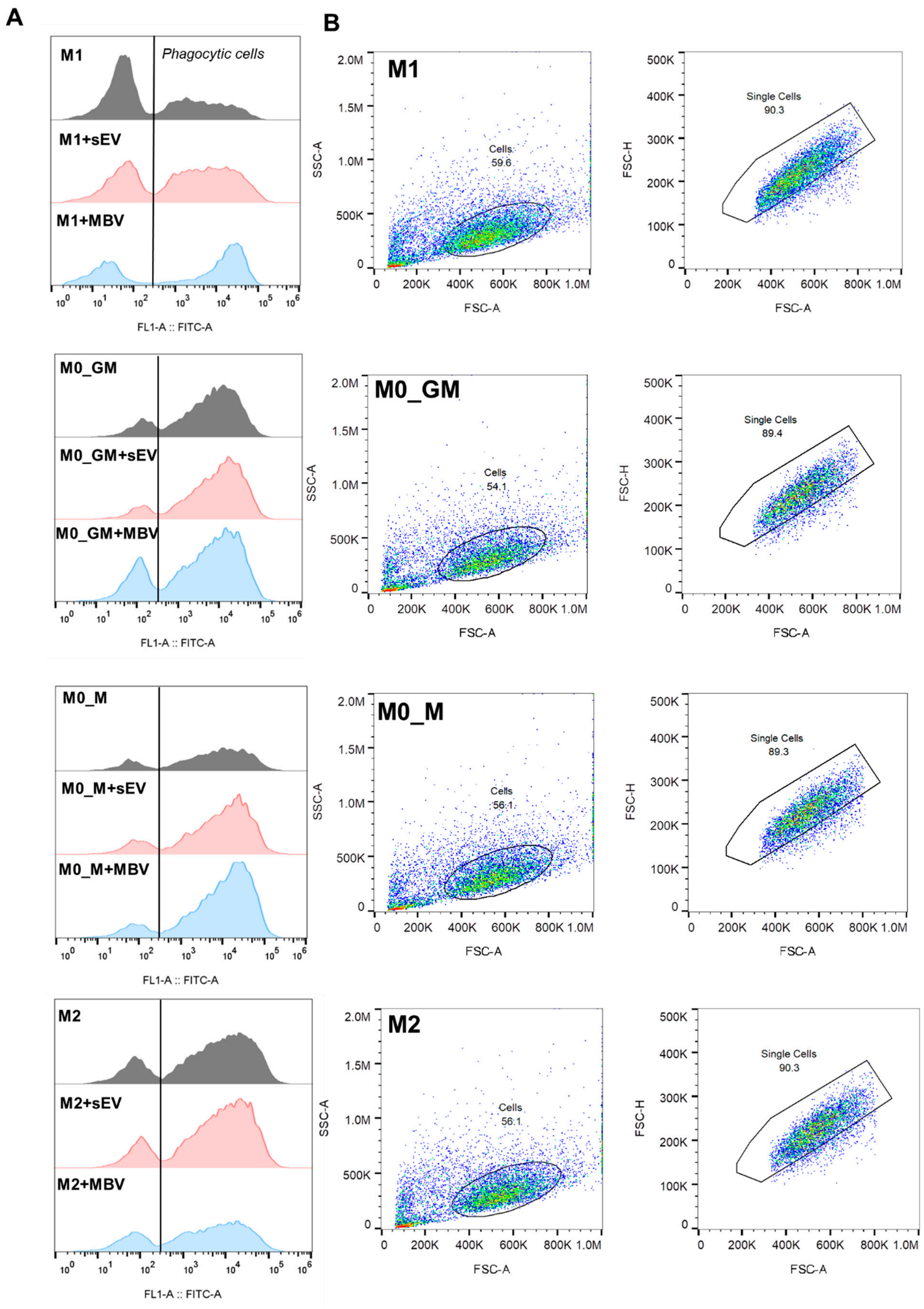

Figure S8. Detection by flow cytometry of phagocytosis of *E. coli* bioparticles after exposure of MDM to sEVs and MBVs UC-MSCs. A – Histograms of FITC fluorescence distribution after incubation of M1, M0_GM, M0_M, and M2 macrophages with FITC-labeled *E. coli* obtained by flow cytometry. The histograms show the percentages of macrophages within the total population that phagocytized FITC-labeled bacteria (located to the right of the vertical line). B – To identify cells and exclude debris, a forward scatter area (FSC-A) versus side scatter area (SSC-A) plot was used. To analyze single cells only, a forward scatter height (FSC-H) versus FSC-A density plot was generated.

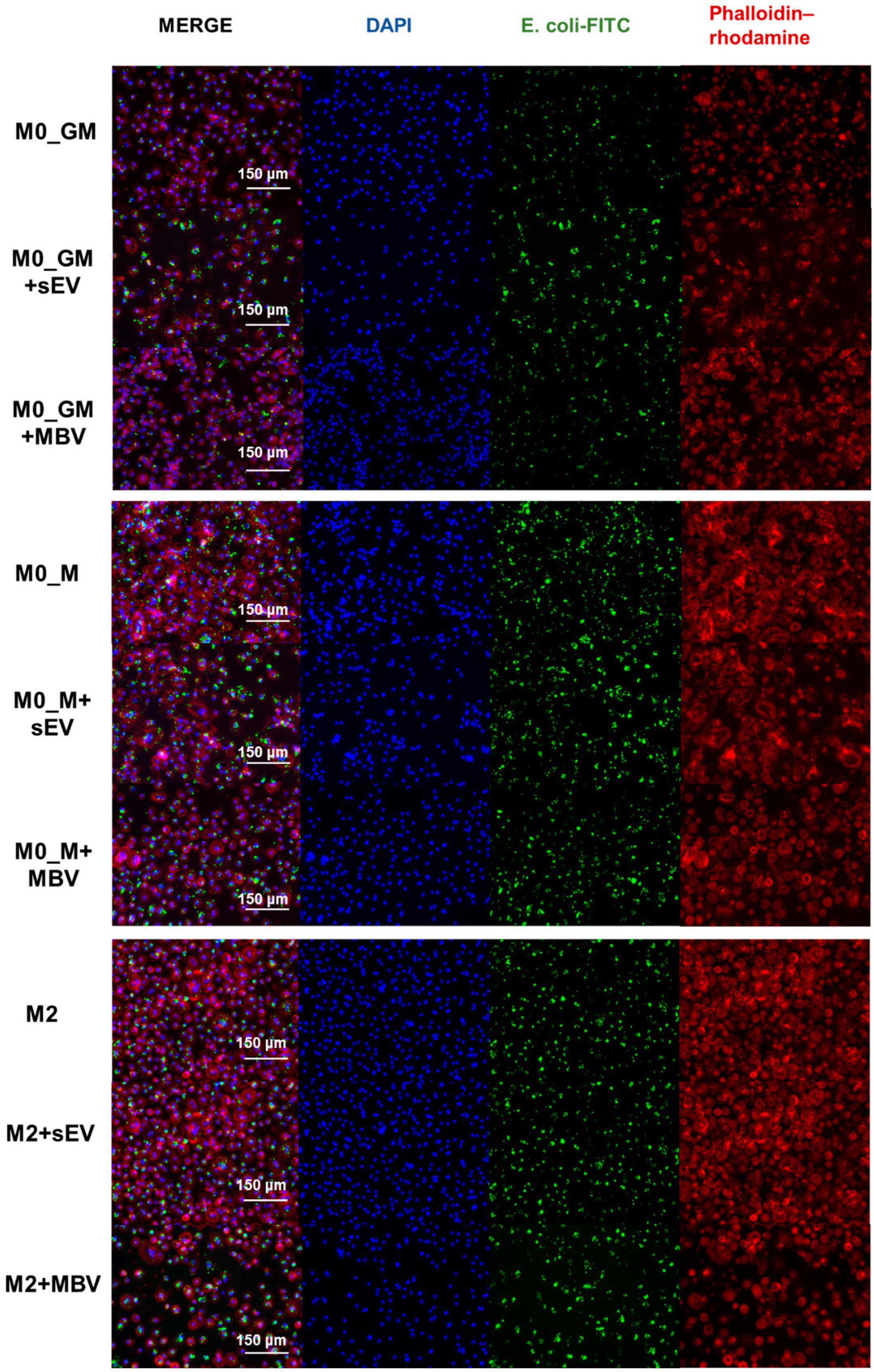

Figure S9. Visualization of phagocytosis of *E. coli* bioparticles by MDM of M0_GM, M0_M, and M2 phenotypes after exposure to sEVs and MBVs UC-MSCs obtained by fluorescence microscopy of fixed human macrophages after incubation with *E. coli* bioparticles at a 10:1 ratio. FITC (green) marks E. coli

bioparticles; phalloidin conjugated with rhodamine (red) stains the actin cytoskeleton; Hoechst (blue) stains the nuclei. Magnification ×20. Scale bar 150 μm.

Micrographs of phagocytosis by MDM of M0_GM, M0_M, and M2 phenotypes after exposure to sEVs and MBVs UC-MSC sdemonstrate trends consistent with those obtained by flow cytometry. M0_M and M2 macrophages showed higher levels of phagocytosis compared to M0_GM and M1 cells. Moreover, after exposure to vesicles, there was a trend toward enhanced phagocytosis in M0_GM macrophages, particularly in the M0_GM+sEVs group.
